# Human eIF3b and eIF3a serve as the nucleation core for the assembly of eIF3 into two interconnected modules: the yeast-like core and the octamer

**DOI:** 10.1093/nar/gkw972

**Published:** 2016-10-19

**Authors:** Susan Wagner, Anna Herrmannová, Darina Šikrová, Leoš Shivaya Valášek

**Affiliations:** Laboratory of Regulation of Gene Expression, Institute of Microbiology ASCR, Videnska 1083, Prague, 142 20, the Czech Republic

## Abstract

The 12-subunit mammalian eIF3 is the largest and most complex translation initiation factor and has been implicated in numerous steps of translation initiation, termination and ribosomal recycling. Imbalanced eIF3 expression levels are observed in various types of cancer and developmental disorders, but the consequences of altered eIF3 subunit expression on its overall structure and composition, and on translation in general, remain unclear. We present the first complete *in vivo* study monitoring the effects of RNAi knockdown of each subunit of human eIF3 on its function, subunit balance and integrity. We show that the eIF3b and octameric eIF3a subunits serve as the nucleation core around which other subunits assemble in an ordered way into two interconnected modules: the yeast-like core and the octamer, respectively. In the absence of eIF3b neither module forms *in vivo*, whereas eIF3d knock-down results in severe proliferation defects with no impact on eIF3 integrity. Disrupting the octamer produces an array of subcomplexes with potential roles in translational regulation. This study, outlining the mechanism of eIF3 assembly and illustrating how imbalanced expression of eIF3 subunits impacts the factor's overall expression profile, thus provides a comprehensive guide to the human eIF3 complex and to the relationship between eIF3 misregulation and cancer.

## INTRODUCTION

Protein synthesis is one of the fundamental cellular processes ensuring genetic information stored in DNA is translated into functional polypeptides. As such, it plays a critical role in nearly all aspects of a cell's life. Even a small disturbance in the timing, spatial distribution and/or fidelity of protein synthesis causes or accompanies many diseases, including various types of cancer (reviewed in (1–3)). Translation is mostly regulated through its initiation phase, which in eukaryotes is modulated by 12 protein initiation factors (reviewed in (4,5)). Of these, eIF3 is the largest and the most complex, in mammals comprising 12 non-identical subunits (named eIF3a-m excluding eIF3j) that together form a complex with a molecular weight of around 800 kDa (6,7). Several lines of evidence suggest that one of the originally identified subunits, eIF3j, which would be the 13th, does not really represent a true eIF3 subunit. It only loosely associates with the eIF3 holocomplex in both yeasts and mammals (8–12) and functionally also seems to be rather divergent (discussed in (13)); hence hereafter we will refer to eIF3j as an eIF3-associated factor.

eIF3 has been implicated in promoting the assembly of the 43S pre-initiation complex (PIC)—which comprises the 40S ribosomal subunit, Met-tRNA_i_^Met^ and several eIFs—and in bridging contacts between the PIC and mRNAs complexed with the eIF4 factors. eIF3 also appears to play a role in scanning and AUG recognition (reviewed in (4)). Besides these general roles in the mechanism of translation initiation, eIF3 has also been demonstrated to regulate translation in a transcript-specific manner (14–16), to promote reinitiation after the translation of short upstream ORFs (17–20), and even to control translation termination and ribosomal recycling (13,21) and stimulate stop codon readthrough (22,23). The involvement of eIF3 in events throughout the translational cycle is consistent with the observation of deregulated eIF3 expression in developmental disorders (24–27), cancer, and other diseases (1–3). And yet, the molecular mechanism of eIF3's involvement in these processes remains unknown, in part owing to the lack of detailed characterization of eIF3 composition and assembly, its interactions with the translational machinery, or the roles of its subunits both in its core functions and in gene-specific translational control.

**Figure 1.**
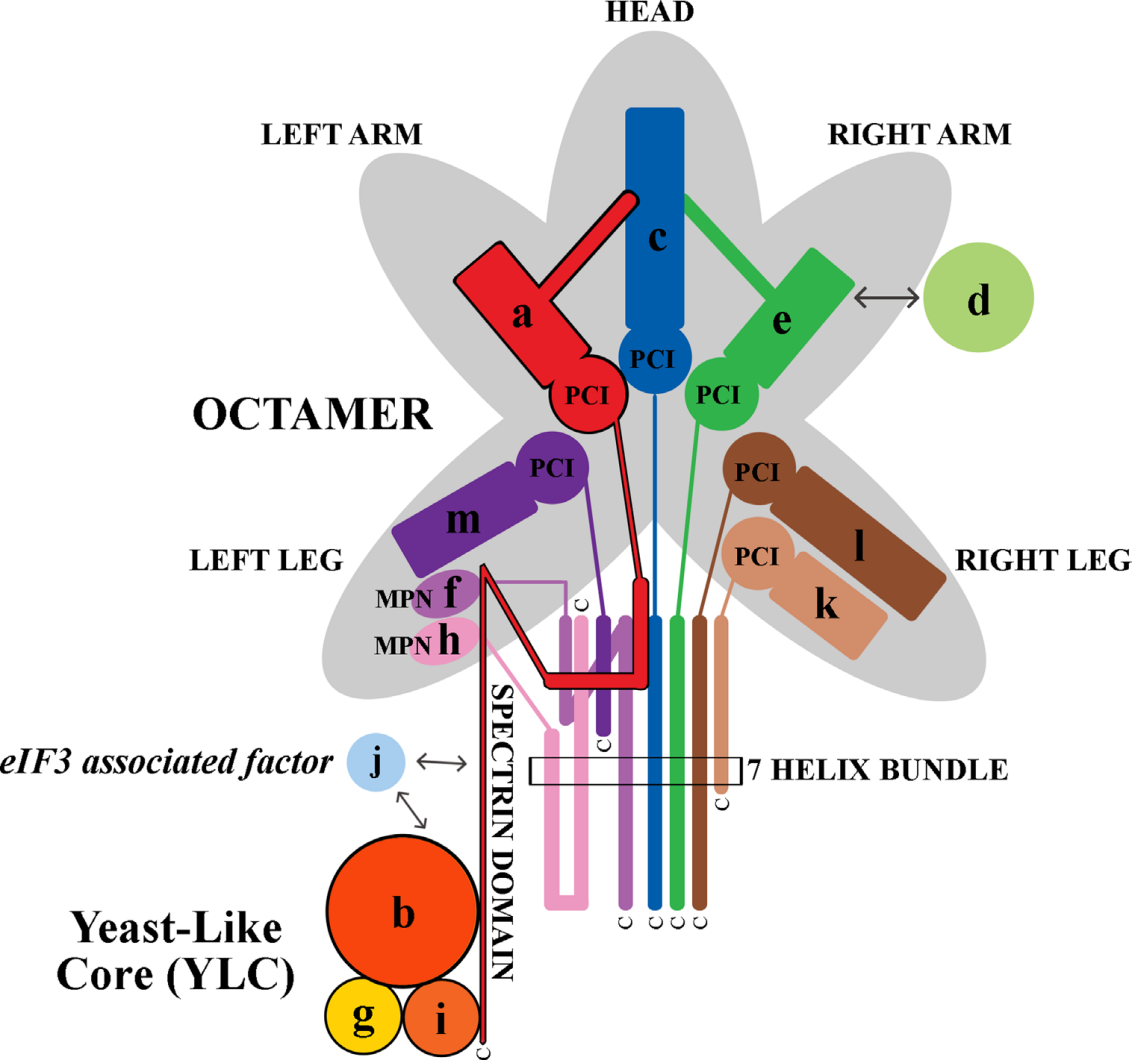
A schematic model of human eIF3 was adapted from (32). The eIF3 subunits forming the PCI/MPN octamer with the anthropomorphic shape are indicated by the grey background. The rectangle marks the seven α-helices involved in formation of the 7-helix bundle (32). The Yeast-Like Core (YLC) comprising the eIF3 subunits a, b, g and i (defined previously (39)) is depicted and so is the eIF3-associated factor eIF3j with arrows indicating its contacts with other eIF3 subunits. The upper right-hand side arrow indicates the interaction between eIF3e and eIF3d that attaches eIF3d to the rest of eIF3 (7,32,40).

Human eIF3 takes the form of an anthropomorphic complex with 5 lobes named the head, right and left arms and right and left legs (28) (Figure 1). Subunits a, c and e form the left arm, head and right arm, respectively. The left leg is composed of subunits f, h and m and the right leg is built from the k and l subunits (29). These eight subunits form a structural scaffold that is shared by the functionally unrelated 19S proteasome lid as well as the COP9 signalosome (30,31) and that is, according to the occurrence of the signature domains contributing to the structure, called the PCI/MPN octamer. Six subunits contain a PCI domain (for **P**roteasome-**C**OP9 signalosome-e**I**F3) and two subunits contain an MPN domain (for **M**pr1-**P**ad1 **N**-terminal). These PCI or MPN structural domains are followed, in each of these eight subunits within all three complexes, by alpha helices that form what is called—at least in the mammalian eIF3 complex—a helical bundle (32)). The remaining four non-octameric subunits (d, b, g and i) are most probably rather flexible; the cryo-EM density for the 12-subunit complex contains little additional density when compared to that for the octamer (33). Despite recent structures illuminating much of the structure of the mammalian eIF3 complex and its interaction with the PIC (32,34,35), how the eight subunits of the octamer are arranged in concert with the remaining four subunits to form the entire complex both on and off the ribosome, and how this ribosome-scale complex is assembled within the cell remain a mystery.

Despite the remarkable conservation of eIF3 composition in most eukaryotes, as well as certain plants and protists (36), the budding yeast *Saccharomyces cerevisiae* is endowed with one of the smallest known eIF3 complexes, consisting of only 5 core subunits: TIF32, PRT1, NIP1, TIF35 and TIF34, respectively, orthologous to the a, b, c, g and i subunits (37); the eIF3-associated factor eIF3j also has an orthologue, HCR1 (9,38). Two Excavata protists, *Trichomonas vaginalis* and *Giardia duodenalis*, have been predicted to have even smaller eIF3 complexes than *S. cerevisiae*, sharing with the budding yeast only the b, c and i subunits (36); however, to our knowledge no experimental data are available to validate these bioinformatic predictions. Taking into account the marked evolutionary conservation of the protein synthesis pathway throughout eukaryotes, it is striking that budding yeast eIF3—itself capable of participating in events throughout the initiation pathway and in other aspects of translation—contains only two of the eight mammalian octameric subunits. These subunits have previously been proposed to represent the structural and functional core of this larger eIF3 complex. These seemingly opposed observations suggest two possibilities. First, it is likely the minimalistic 5-subunit core of the budding yeast eIF3 that constitutes the basic functional unit sufficient to support life in a unicellular organism such as *S. cerevisiae*. Second, the added subunit complexity of other eIF3 factors likely represents its robust participation in translation regulation. In strong support of this hypothesis, we previously described a stable subcomplex composed of the a, b, g and i subunits of human eIF3 that closely resembles the minimal budding yeast complex and demonstrated that it performs the basic functions of eIF3 in HeLa and HEK293 cells (39). Moreover, others have reported that a similar complex (even containing the eIF3c subunit [NIP1 in yeast]) can form *in vitro* at increased levels of ionic strength (7), as well as in *Neurospora crassa*, a fungus whose eIF3 displays the complex subunit composition observed in mammals (40). In accordance with these observations, we will refer to this human a-b-g-i subcomplex as the **Y**east-**L**ike **C**ore (YLC).

Altered expression levels of several eIF3 subunits have been observed in various types of cancer (reviewed in (1)). The majority of these studies monitored the levels of only one subunit of eIF3. However, recent investigations have shown that changes in the expression of one eIF3 subunit can affect the expression of other eIF3 subunits (see for example (26,39,41)). As we have demonstrated, this may lead to the formation of eIF3 subcomplexes capable of performing a subset of the eIF3 repertoire of functions (39). It is therefore important to understand the entire network of physical and functional interactions among the eIF3 subunits in order to reveal how each subunit affects the stability and expression of its counterparts.

To this end, we have complemented our previous siRNA analysis of human eIF3 by knocking down the remaining 10 subunits of eIF3 in HeLa cells. With the help of co-immunoprecipitation and Western blot analyses, we show here that several stable eIF3 subcomplexes can form *in vivo* when the expression of certain eIF3 subunits is compromised. We also demonstrate the impact of individual eIF3 knock-downs on cell fitness, translation initiation rates and the stability of the remaining, non-targeted eIF3 subunits. Based on our results we propose *in vivo* assembly and disassembly pathways for human eIF3 and describe an array of expression dependencies among the eIF3 subunits.

## MATERIALS AND METHODS

### Whole cell extract (WCE) preparation and transfection

HeLa cells were grown at 37°C and 5% CO_2_ in 24-well plates, Ø10 cm or Ø15 cm dishes in DMEM (Sigma, cat # D6429) supplemented with 10% FBS (Sigma, cat # F7524). Twenty four hours after seeding, cells were transfected with the ON-TARGETplus siRNA cocktail system from Dharmacon at a final concentration of 5 nM. Catalog numbers for all siRNAs used in this study are listed in Supplementary Table S1. INTERFERin (Polyplus, cat # 409) was used as a transfection reagent and transfection was performed according to the vendor‘s instructions. Cells were harvested 3 days after transfection, as described previously (39), except that the Tris-based lysis buffer was substituted with a HEPES-based buffer (10 mM HEPES (pH 7.5), 62.5 mM KCl, 2.5 mM MgCl_2_, 1 mM DTT, 1 mM PMSF, 1μ g/ml Aprotinin, 1 μg/ml Leupeptin, 1 μg/ml Pepstatin, mini Complete EDTA-free (Roche) 1 tablet/5 ml, 1% Triton X-100).

### Western blotting and quantification

All samples were resolved using SDS-PAGE followed by western blotting. All primary antibodies used in this study are listed in Supplementary Table S2. The signals obtained from all eIF3 antibodies are shown on an uncropped gel and with size markers indicated in Supplementary Figure S1. The specificity of each band was determined by (i) its mobility at the expected position on the SDS-PAGE as predicted by its molecular weight, (ii) the fact that it specifically co-immunoprecipitates with the rest of eIF3 (see our eIF3b and eIF3f Co-IP experiments here and in (39) and (iii) the fact that it specifically co-sediments with PICs in sucrose gradients (39). The western signal was developed using SuperSignal West Femto Maximum Sensitivity Substrate from Thermo Scientific (cat # 34096) and detected in a G-Box imager from Syngene using a series of varying exposure times. Signals were processed with Quantity One (BioRad). Only signals from the same strips and with the same exposure times were compared. If not stated otherwise, reported values represent the mean of at least five individual experiments. The resulting values were normalized as indicated in the corresponding figure legends.

### Co-Immunoprecipitation assays

eIF3 complexes were immunoprecipitated from WCEs, the preparation of which is described above, using GammaBind G Sepharose (GE Healthcare, cat # 17-0885-01) with either anti-eIF3b (Santa Cruz, cat # sc-16377) or anti-eIF3f (kind gift of Dr Hiroaki Imataka) primary antibodies. The detailed CoIP protocol was reported previously (39). To visualize western signals particularly from the eIF3f-CoIP, a protein-A linked to peroxidase (GE Healthcare, cat # NA9120) had to be used because of the rabbit origin of the eIF3f antibodies.

### PARP cleavage detection

WCEs from cells treated with different siRNAs were subjected to western blotting to detect the presence of cleaved poly(ADP-ribose)polymerase-1 (PARP-1). PARP-1 is a target of caspase-3 and -7 and is intact in cells that have not entered apoptosis. As a positive control, apoptosis was induced by incubating non-transfected cells with 1 μM staurosporine (Cell Signaling, cat # 9953) for 4 h at 37°C. DMSO was used as control.

### MTT assay and polysome profile analysis

MTT assay and polysome profiling were carried out as described previously (39).

### RNA isolation, reverse transcription and qPCR

Total RNA was isolated using RNA Blue reagent (Top Bio, cat # R013) 72 h post-transfection according to the manufacturer's instructions. After DNase I digestion (NEB, cat # M0303L), cDNA was synthesized using the High-capacity cDNA reverse transcription kit (Applied Biosystems, # 4368813). qPCR was performed using 5× HOT FIREPol EvaGreen qPCR Mix Plus (Solis BioDyne # 08-25-00020). The obtained data were normalized to B2MG mRNA levels; at least two individual experiments were performed. qPCR primers are listed in Supplementary Table S3.

### mRNA binding analysis

Polysomal gradients were run as described previously (39), with the exception that a 10–50% sucrose gradient was used here. 49 fractions were collected and 50 ng of *in vitro*-transcribed yeast RPL41 mRNA was added to each fraction as the RNA spike prior to further manipulation. Subsequently, SDS was added to each fraction to a final concentration of 1%, and two successive acid phenol:chloroform (5:2) extractions were carried out (first at 65°C and the second at the room temperature), followed by one chloroform:isoamylalcohol (25:1) extraction. RNA was then precipitated with ethanol in the presence of 300 mM sodium acetate at −20°C. DNaseI digestion, reverse transcription and qPCR were carried out as described above. The same fraction of each sample was used for the reverse transcription and qPCR, and Cq values were normalized to the RNA spike.

## RESULTS

### ‘Knockin’ on eIF3's door’—individually downregulating three octameric and all non-octameric eIF3 subunits produces deleterious effects on the rate of translation initiation and the overall proliferation of human cells

To study the assembly and function of the entire human eIF3 complex *in vivo* we employed RNA interference (using ON-TARGETplus siRNAs listed in Supplementary Table S1) and individually knocked down each of the 12 subunits of eIF3, as well as the eIF3-associated eIF3j in HeLa cells. We previously applied this strategy to study the functions of the eIF3a and c subunits and eIF3j (39). Here, we present our results for the remaining 10 eIF3 subunits (b, d, e, f, g, h, i, k, l and m) and compare them with our earlier findings (39).

As before, all experiments described here were carried out three days after the subunit-specific siRNA transfection of HeLa cells, at which point the protein levels of each targeted subunit were decreased by ∼80–90% (Tables 1–3). Importantly, none of the knocked down cultures had entered apoptosis at this time point, as judged by the poly(ADP-ribose)polymerase-1 (PARP-1) cleavage assay (Supplementary Figure S2), even though approximately half of the knock-downs did not recover after the siRNA treatment and the cells eventually died (see below).

**Table 1.** Quantification of experiments shown in Figure 3 and Supplementary Figure S4

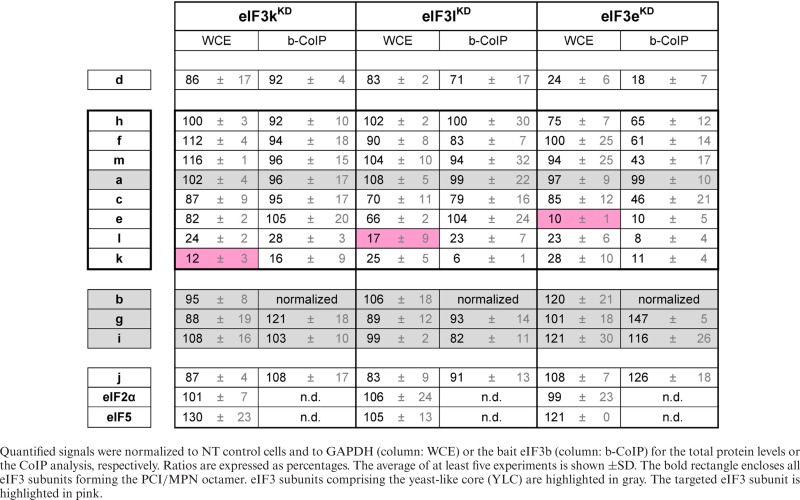						

**Figure 2.**
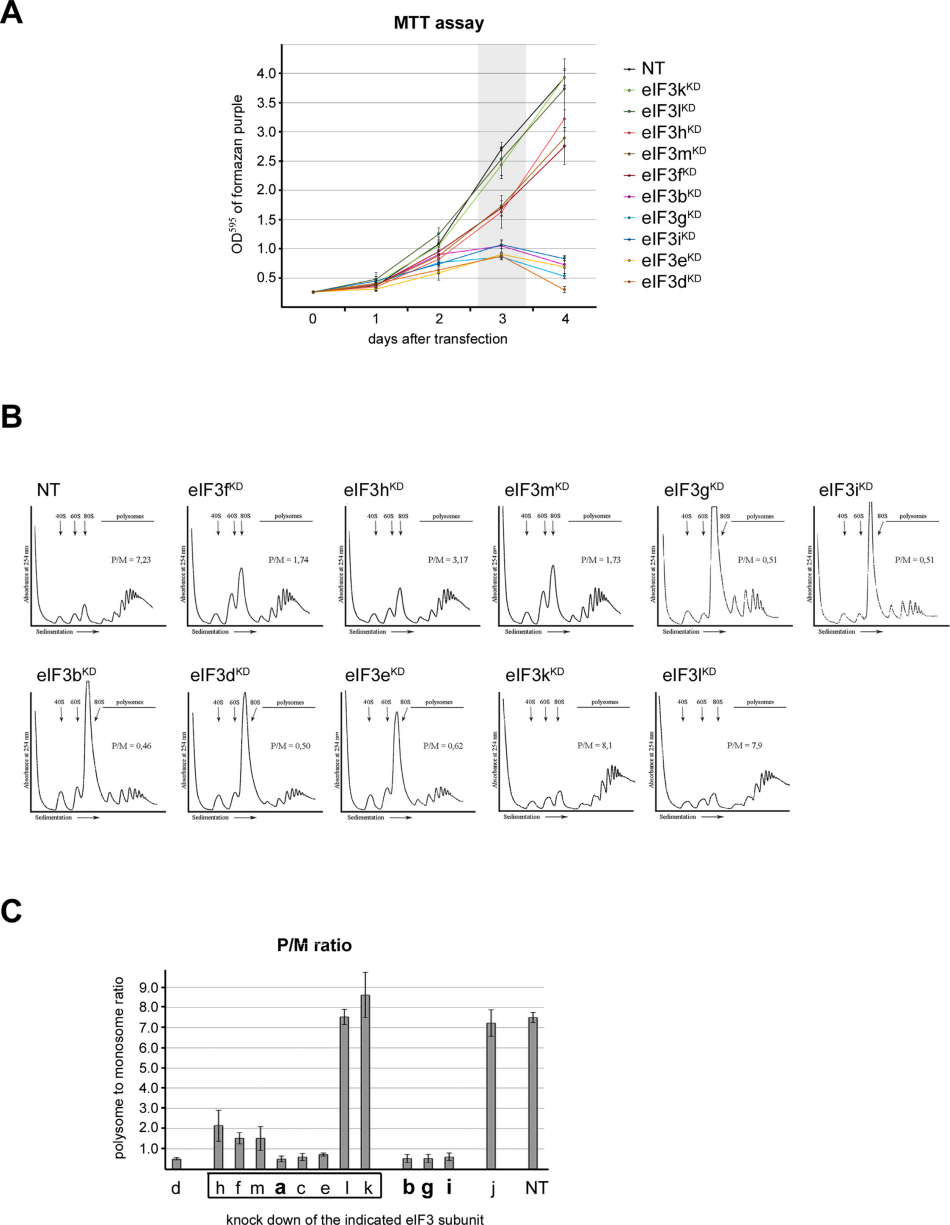
Downregulation of the YLC subunits (b, g, i), the non-octameric eIF3d or the octameric subunits a, c, and e has deleterious effects on initiation rates and the overall cell proliferation. (**A**) The effect of indicated knock-downs of individual eIF3 subunits on cell proliferation of HeLa cells was assessed by the MTT assay 1 to 4 days post-transfection. The panel represents the results from three independent experiments ± SD. The grey box indicates the time point at which all follow-up analyses were conducted. (B and C) The effect of the indicated knock-downs on translation initiation rates was assessed by polysome profile analyses 3 days post-transfection. One example profile for each knock-down is shown in panel (**B**). (**C**) The plot shows the polysome to monosome ratio (P/M) from three independent experiments ± SD. Results for eIF3a^KD^, c^KD^ and j^KD^, which were shown in (39), are included as well, for the sake of completeness. The octameric subunits are indicated by the rectangle; the YLC subunits are shown in bold.

We first measured the proliferation and overall fitness of each cell population over several days using the MTT assay, which follows the reduction of yellow thiazolyl-tetrazolium-bromide (MTT) to purple formazan (Figure 2A). Inhibited proliferation, which leads to a lower number of cells and reduces the fitness of these surviving ones, results in an overall decrease in the reduction of MTT to formazan. All tested knock-downs fall into three groups, according to the severity of their growth defect. Downregulation of the right leg subunits eIF3k and eIF3l had no effect on overall cell fitness (Figure 2A), indicating that these subunits are either not essential or that the residual protein is sufficient to support wild-type growth. A similar effect was previously reported for the knock-down of the eIF3-asssociated factor eIF3j (39). Downregulation of the left leg subunits of the octamer (f, h and m) partially diminished cell fitness by day 3, but cell growth improved with time and cultures ultimately recovered after siRNA treatment; this effect was most rapid for the eIF3h knock-down (eIF3h^KD^), which recovered by day 4 (Figure 2A). In contrast to these effects, individual knock-down of either the non-octameric subunits (b, g, i and d) or of the three subunits composing the head and both arms of the octamer—a and c as demonstrated in (39) and eIF3e—strongly diminished cell fitness, an effect from which cells were unable to recover (Figure 2A). These results may suggest that the octamer head with both arms (c, a, and e) and the YLC module (comprising the a, b, g and i subunits) can form, together with eIF3d, a minimal eIF3 complex via the shared eIF3a subunit that is partially capable of performing the core functions of the eIF3 holocomplex.

Accordingly, polysome profile analysis of these cells demonstrated that knock-down of eIF3 subunits producing the most severe growth defects also similarly produced the largest observed decreases in the ratio of polysomes to monosomes (P/M), when compared to cells treated with non-targeting siRNA (Figure 2B and C). The P/M ratio reflects translation rates; the high P/M ratios observed upon knock-down of eIF3k^KD^, eIF3l^KD^ and eIF3j^KD^, whose numbers do not differ from control cells, reflect efficient translation (Figure 2C). In contrast, we observe a greater than 70% drop in the P/M ratios for cells in which the left leg f, h and m subunits have been knocked-down. In fact, these effects appear more dramatic than those we observe on the growth of these cells (Figure 2A). This suggests that the relationship between the proliferation and translation rates is not linear. In other words, that the relatively low P/M ratio observed upon knock-down of eIF3f, h and m is sufficient to support significant proliferation rates, but perhaps not to maximize the proliferation rate under most optimal conditions. In fact, we have observed that the P/M ratio can vary dramatically (3 ≤ P/M ≤ 8) in NT cells exhibiting only modest changes in confluency (unpublished observation).

### The octameric right leg subunits eIF3k and eIF3l impact their mutual expression but are dispensable for the integrity of the rest of the eIF3 complex

In our pilot study, we observed that protein downregulation of specific eIF3 subunits, like eIF3a^KD^ and eIF3c^KD^, leads to simultaneous downregulation of other, non-targeted eIF3 subunits (39). We showed that this downregulation occurs exclusively on their protein levels and also that other eIFs such as eIF2 or eIF5 are not affected. Hence, we next wished to investigate this phenomenon for the remaining 10 eIF3 subunit knock-downs.

First, we examined the effects of each individual knock-down on the mRNA levels of all 12 eIF3 subunits. Consistent with our previous results, only the mRNA levels of the targeted subunit were reduced; the mRNA levels of the remaining 11 non-targeted subunits were virtually unchanged (Supplementary Figure S3 and (39)).

**Figure 3.**
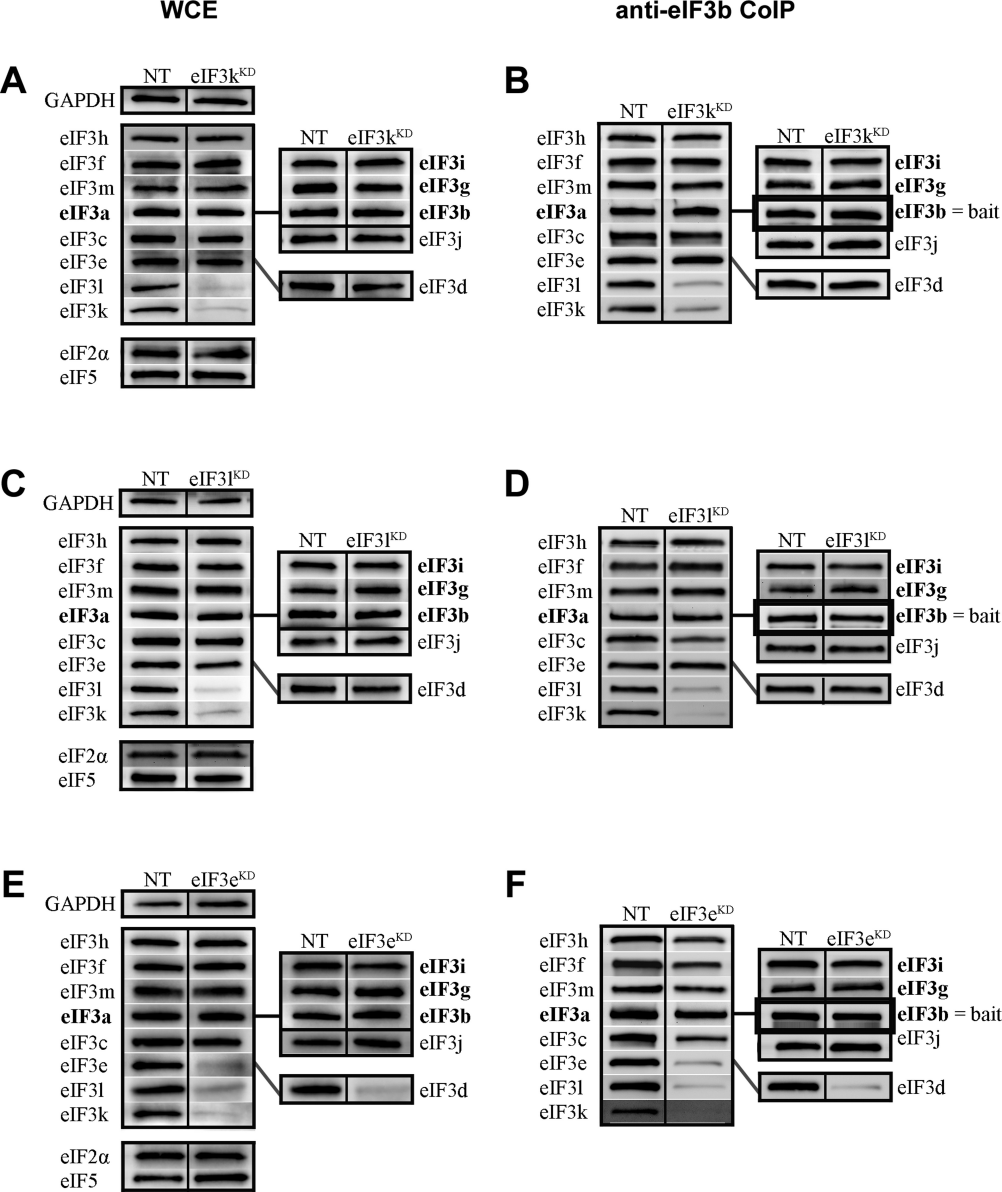
The octameric right leg subunits eIF3k and eIF3l impact their mutual expression and are dispensable for the integrity of the rest of eIF3, and the octameric right arm subunit eIF3e stabilizes binding of the right leg subunits (k, l) and eIF3d to the octamer, as well as the octamer attachment to the YLC. Protein levels of all eIF3 subunits and other eIFs upon knock down of either eIF3k, l or e were determined by Western blotting (**A, C, E**, respectively). GAPDH was used as a loading control. To assess the integrity of eIF3, the anti-eIF3b co-immunoprecipitation experiments were carried out for each knock-down and the immunoprecipitated eIF3 subunits along with eIF3b were detected by Western blotting (**B, D, F**). The octameric eIF3 subunits are arranged at the left-hand side of each panel and non-octameric subunits are at the right-hand side. The YLC subunits are grouped and highlighted in bold. Quantifications of at least five independent experiments with standard deviations are shown in Supplementary Figure S4 and Table 1.

In contrast, transfection with siRNA against eIF3k led to simultaneous, robust downregulation of eIF3l protein levels, and *vice versa* (Figure 3A and C, Supplementary Figure S4A and C, and Table 1). The remaining 10 eIF3 subunits were well expressed (more than 80% of the levels observed in control cells treated with non-targeting siRNA), with only the eIF3e (right arm) and eIF3c (head) subunits, which are the closest subunits to eIF3l in the schematic shown in Figure 1, displaying a slightly greater (∼30-35%) decrease of their protein levels in eIF3l^KD^ cells (Figure 3A and C, Supplementary Figure S4A and C, and Table 1).

To determine if the 10 eIF3 subunits unaffected by eIF3k or eIF3l knock-down can still form a stable complex, we co-immunoprecipitated the eIF3 subunits using eIF3b as bait. In untreated cells or cells treated with non-targeting siRNA, anti-eIF3b antibodies efficiently pull down all 12 eIF3 subunits and the eIF3j factor, as reported previously (39) (Supplementary Figure S5A). Neither the selected small ribosomal proteins nor eIF3 binding partners eIF2 and eIF5 were recovered, even though based on our previous yeast studies (9,42) the latter two eIFs might have been expected to co-purify with eIF3. In eIF3k^KD^ and eIF3l^KD^ cells, all 10 well-expressed eIF3 subunits are efficiently pulled down with anti-eIF3b (Figure 3B and D; Supplementary Figure S4B and D, and Table 1).

To complement these experiments, we additionally co-immunoprecipitated the eIF3 subunits using eIF3f as bait. As we observe when using eIF3b as bait, anti-eIF3f antibodies pull down all 12 eIF3 subunits and eIF3j from control cells transfected with non-targeting siRNA (Supplementary Figure S5B). This antibody pulls down small amounts of eIF2 but virtually no eIF5 or small ribosomal proteins. Because eIF3f is an integral part of the octamer (whereas eIF3b is not), this approach balances the eIF3b pulldowns because the former pulls the complex down via the YLC, whereas the latter pulls it down via the octamer. As expected, anti-eIF3f antibodies pulled down all but eIF3k and l subunits in eIF3k^KD^ cells (Supplementary Figure S6A and B). Collectively, these results strongly suggest that the k and l subunits of eIF3 are dispensable for the formation of a stable and fully functional eIF3 complex. This in turn explains the fact that they are not essential for normal cell proliferation.

### The eIF3e subunit within the right arm of the octamer stabilizes binding of the right leg subunits (k, l) and eIF3d to the octamer and supports the octamer attachment to the YLC

We next tested the effects of eIF3e downregulation on the integrity of the eIF3 complex. siRNA knockdown of eIF3e resulted in simultaneous, severe downregulation of the protein levels of subunits within the entire right side of the eIF3 octamer body: the right leg subunits eIF3k and eIF3l, as well as the non-octameric eIF3d subunit (Figure 3E, Supplementary Figure S4E, and Table 1). The latter is consistent with recent studies where eIF3e was proposed to connect eIF3d to the rest of the octamer (32,40). All other eIF3 subunits were unaffected by eIF3e downregulation. Interestingly, eIF3b CoIP experiments revealed that simultaneous downregulation of the former subunits impacts the integrity of the eIF3 complex, as only the YLC subunits (a, g and i) and eIF3j efficiently co-purified with the eIF3b bait. In contrast, the subunits within the octamer left leg (f, h and m) as well as the eIF3c subunit within the head co-purified with the YLC module at reduced levels (by ∼45 - 65%), despite their protein levels not being affected (Figure 3F, Supplementary Figure S4F, Table 1). These data indicate that at least two partial subcomplexes form: 1) the YLC module with eIF3j alone and 2) a complex containing the YLC module, eIF3j, and the head and left limbs of the octamer (namely eIF3c, f, h, m and shared eIF3a). Indeed, using anti-eIF3f as the bait, we confirmed the existence of this second subcomplex, as it was pulled down with 100% efficiency compared to cells treated with non-targeting siRNA (Supplementary Figure S6C and D). The fact that we did not observe increased amounts of the eIF3c-f-h-m subunits co-precipitating with anti-eIF3f antibodies in these knock-down cells suggests that the eIF3c-f-h-m octameric subunits do not form a subcomplex stable enough to survive our purification procedure.

Similarly, we previously observed that two distinct eIF3 subcomplexes can be purified from eIF3c^KD^ cells (39). Knocking down the eIF3c head subunit resulted in simultaneous downregulation of the right limb subunits e, k, and l, as well as the non-octameric eIF3d subunits, breaking eIF3 into two subassemblies: the YLC with eIF3j and the left leg f-h-m trimer.

Taken together, these findings suggest that eIF3e stabilizes the attachment of the k and l subunits of the right leg to the rest of the octamer. Moreover, they support the previous observation that eIF3e directly connects the d subunit to the eIF3 complex (32,40). Finally, these results indicate that besides the left arm subunit eIF3a—which is shared by both the octamer and the YLC modules—both the eIF3c subunit and to a smaller degree also the eIF3e subunit support the attachment of the left leg subunits (f, h, m) to the YLC module via their attachment to the eIF3a subunit.

### The eIF3f and m subunits of the octamer left leg are, together with eIF3c and eIF3a, the key building blocks of the PCI/MPN octamer

**Figure 4.**
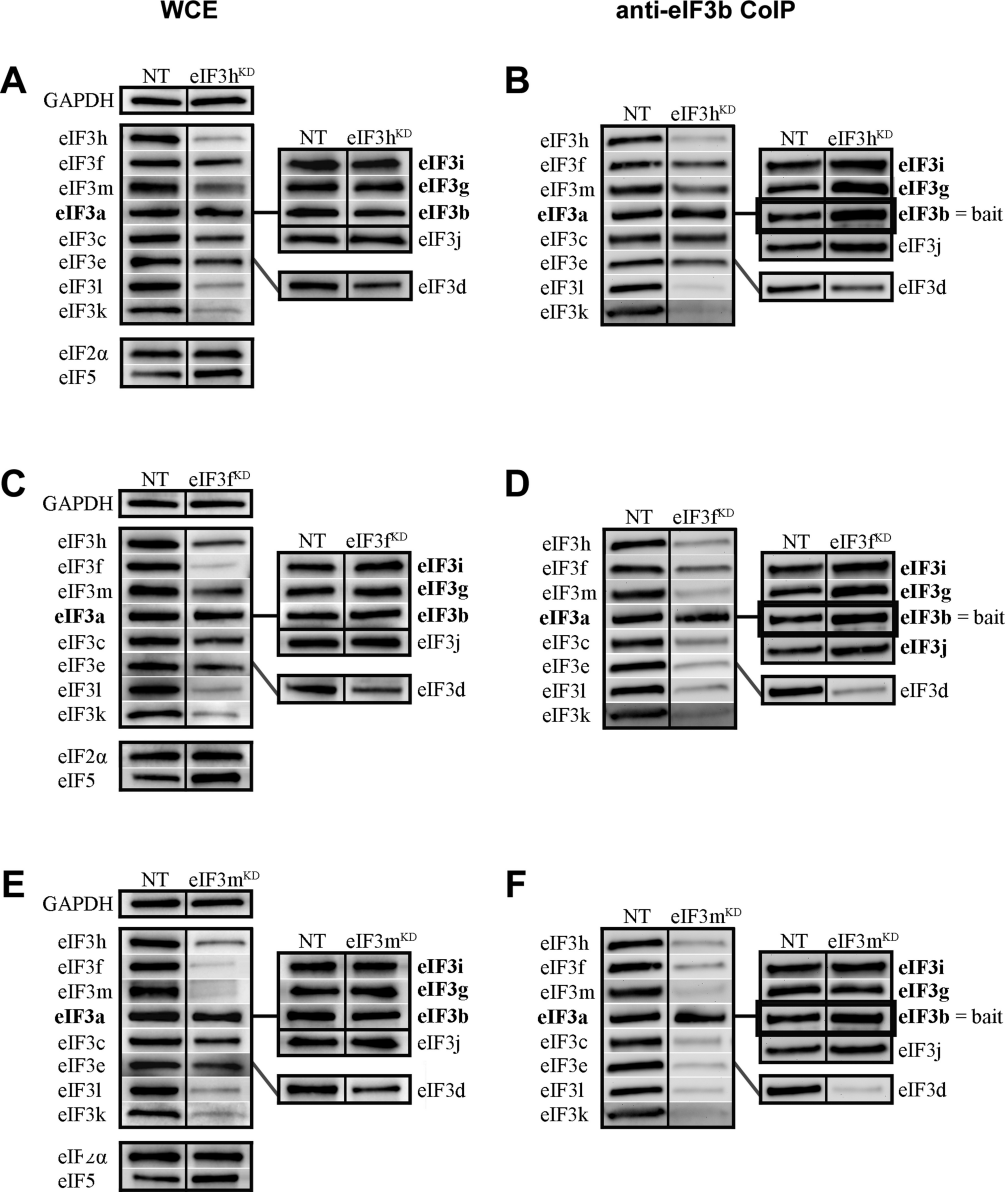
The octameric left leg subunits eIF3f and m represent, together with eIF3c and eIF3a, the key building blocks of the PCI/MPN octamer. Similar to Figure 3 except that knock-downs of either eIF3h (**A** and **B**), f (**C** and **D**) or m (**E** and **F**) were analyzed. Quantifications of at least five independent experiments with standard deviations are shown in Supplementary Figure S7 and Table 2.

Moving to the subunits of the octamer left leg, we observe that targeted siRNA of the eIF3h subunit within the octamer left leg strongly reduces the protein levels of the right leg subunits eIF3k and eIF3l, to ∼30% of those observed in control NT cells (Figure 4A, Supplementary Figure S7A, and Table 2). The protein levels of the remaining octameric subunits eIF3a, c, e, f and m and the non-octameric eIF3d subunit were reduced less dramatically (by ∼20-40%), with eIF3a being the least affected (Figure 4A, Supplementary Figure S7A, and Table 2).

**Table 2.** Quantification of experiments shown in Figure 4 and Supplementary Figure S7

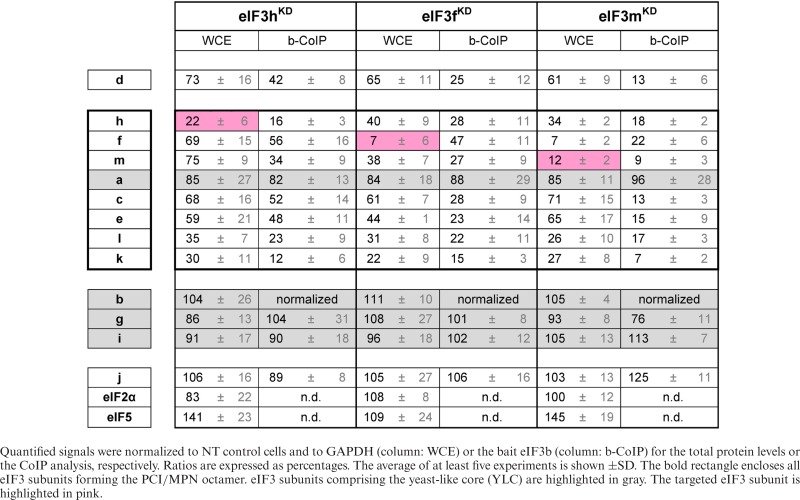						

Co-immunoprecipitation experiments with anti-eIF3b revealed again that two partial subcomplexes can form: 1) the YLC module with eIF3j alone and 2) the YLC module with eIF3j, eIF3d and the octamer lacking both its right leg (3k and l) and a part of the left leg (3h). The former complex forms with very high efficiency while the latter complex occurs at ∼40–50% of the levels observed for the former (Figure 4B, Supplementary Figure S7B, and Table 2). Co-immunoprecipitation experiments with anti-eIF3f confirmed the existence of the larger 9-subunit complex, which was pulled down at ∼60% of efficiency observed from control NT cells (Supplementary Figure S6E and F). These findings suggest that eIF3h collaborates with eIF3e to attach the right leg subunits eIF3k and l to the octamer.

siRNAs directed against either eIF3f or eIF3m affect the expression and integrity of the eIF3 complex similarly. Protein levels of both the left (f, h, m) and right (k, l) legs were strongly decreased (down to 20–40% for both knock-downs). Notably, eIF3f protein levels were more severely compromised in eIF3m^KD^ cells than in the reciprocal case (eIF3m levels in eIF3f^KD^ cells) (Figure 4C and E, Supplementary Figure S7C and E, and Table 2). In contrast, the levels of the right arm subunit eIF3e were more severely downregulated in eIF3f^KD^ cells (by ∼55%) than in eIF3m^KD^ cells(by ∼35%); we are currently investigating the origin of these effects. Protein levels for the eIF3c (octamer head) and non-octameric eIF3d subunits were reduced less dramatically (by 30–40%). The levels of the YLC subunits were largely unaffected.

Despite these differences, the eIF3b CoIP experiments produce similar results for both eIF3f and eIF3m knock-downs and further resemble our earlier data obtained with eIF3c^KD^ cells (39). With the exception of the YLC module and eIF3j, all eIF3 subunits co-purify with eIF3b at significantly reduced levels from (∼15–30% of control cell levels), from either eIF3f^KD^ or eIF3m^KD^ cells (Figure 4D and F, Supplementary Figure S7D and F, and Table 2). Interestingly, there is a slight enrichment of the targeted subunit, eIF3f, from eIF3f^KD^ cells, which we cannot explain at present. Hence in the absence of eIF3f and eIF3m, the a, c and e subunits are unable to form a stable upper body subcomplex of the octamer connected to the YLC. Together with our earlier experiments with eIF3a^KD^ and eIF3c^KD^ (39), these results suggest that the subunits of the head (c), left arm (a) and left leg (m and f) of eIF3 are critically required for formation of the eIF3 octamer module.

### The eIF3b subunit of the yeast-like core is required for formation of the complete eIF3 holocomplex

**Figure 5.**
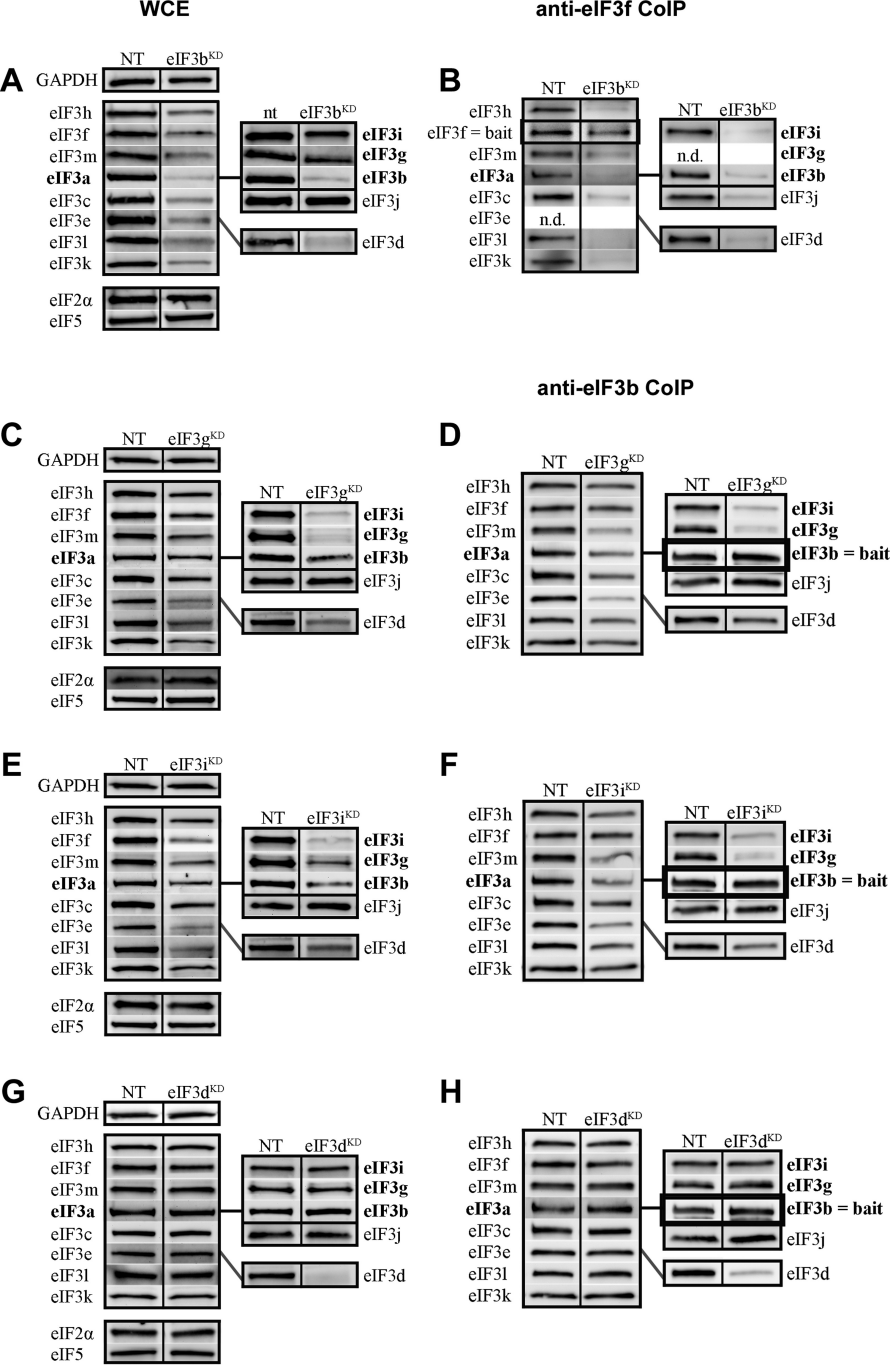
The YLC subunits eIF3b, g and i are required for a stable formation of the eIF3 holocomplex, whereas eIF3d is dispensable for stable complex formation. Similar to Figure 3 except that knock-downs of either eIF3b (**A** and **B**), g (**C** and **D**), i (**E** and **F**) or d (**G** and **H**) were analyzed. The integrity of the eIF3 complex in the eIF3b knock-down was examined by the anti-eIF3f CoIP analysis instead of anti-eIF3b (panel B). The signal of our anti-eIF3e and anti-eIF3g antibodies could not be clearly visualized in the anti-eIF3f CoIPs, because of the heavy chain of eIF3f antibodies of the rabbit origin migrating at the same size (in spite of using the protein-A linked peroxidase; n.d. = not determined). Quantifications of at least five independent experiments with standard deviations are shown in Supplementary Figure S8 and Table 3.

The effects of eIF3b downregulation appear to be more general. Targeted siRNA downregulation of eIF3b led to a broad-spectrum, pattern-less downregulation of all eIF3 subunits to ∼40–70% of the levels observed in control cells, with the exception of eIF3i and the eIF3j factor, which are not significantly reduced (Figure 5A, Supplementary Figure S8A, and Table 3). Consistent with this, all eIF3 subunits co-precipitate with eIF3f at dramatically reduced levels from these cells, reflecting the severe instability of the whole eIF3 complex and the general effect of eIF3b downregulation (Figure 5B, Supplementary Figure S8B, and Table 3). This intriguingly suggests that the eIF3 octamer does not form *in vivo* without the non-octameric eIF3b subunit and that eIF3b is crucial, as it is in yeast (42,43), for stable formation of the entire eIF3 complex, and therefore for maintaining the optimal protein levels of the majority of human eIF3 subunits.

**Table 3.** Quantification of experiments shown in Figure 5 and Supplementary Figure S8

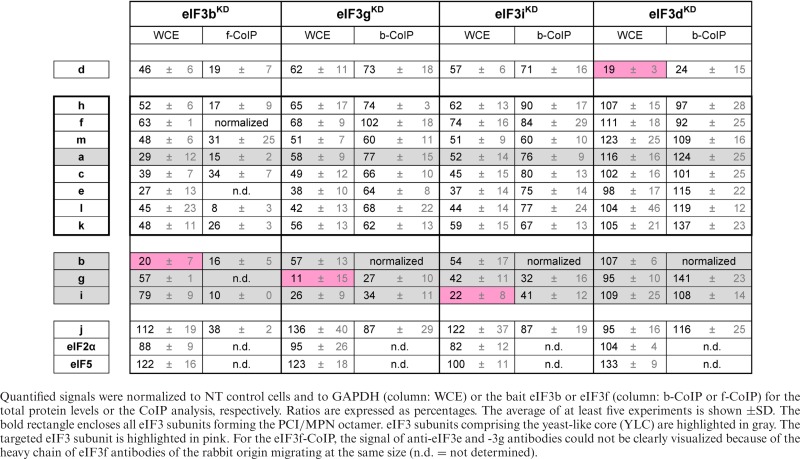								

### The yeast-like core eIF3g and eIF3i subunits mutually promote their own attachment to the eIF3 complex

We next tested the effects of downregulating the i and g subunits of the YLC. When eIF3g was targeted by siRNA, protein levels of eIF3i strongly decreased to 26%. However, knocking down eIF3i did not have the reciprocal effect on eIF3g protein levels, which were affected less dramatically in eIF3i^KD^ cells (Figure 5C and E, Supplementary Figure S8C and E, and Table 3). Because these subunits bind to each other (7) while eIF3i binds to both eIF3a and eIF3b and eIF3g interacts only with eIF3b within the eIF3 complex (44) (Figure 1), it is possible that the unique network of interactions in which each subunit participates underlies these distinct effects. Alternatively, these effects might be a result of eIF3i knock-down being less efficient than that of eIF3g. High-resolution structural studies will likely be required to explain these effects completely. Beyond this difference, both knock-downs resulted in a broad-based reduction (between ∼30 and 60%) in the levels of the remaining 10 subunits (excluding the eIF3j factor) similar to that observed in eIF3b^KD^ cells. In the case of both eIF3i^KD^ and eIF3g^KD^, all eIF3 subunits except for eIF3g and eIF3i co-immunoprecipitated with anti-eIF3b or anti-eIF3f at levels ranging between ∼60 and 90% (Figure 5D and F, Supplementary Figure S8D and F, and Table 3) or ∼40–70% (Supplementary Figure S9A–D), respectively. Direct comparison of the anti-eIF3f Co-IP experiments from eIF3b^KD^, eIF3i^KD^, and eIF3g^KD^ cells clearly underscores that knocking down eIF3b produces the most severe impact on the integrity of the eIF3 complex (compare Figure 5B with Supplementary Figure S9A and C). This implies that: 1) the eIF3 complex can form in the absence of the g and i subunit; 2) they promote binding of each other to the eIF3 complex and 3) they further promote the critical role of eIF3b in organizing the eIF3 holocomplex, possibly by stabilizing assembly of the evolutionary conserved YLC, whose formation may precede the formation of the octamer. Taken together with the eIF3a^KD^ and eIF3b^KD^ data, we propose that eIF3b functions, together with the major scaffolding subunit eIF3a, as the nucleation core of the entire eIF3 complex: the YLC nucleates around eIF3b whereas the octamer nucleates around eIF3a. In the absence of these two subunits, neither the YLC nor the octamer can form.

### The non-octameric subunit eIF3d affects neither the expression nor the integrity of eIF3 but is nonetheless essential for cell proliferation

Like the eIF3-associated factor eIF3j (39), eIF3d is the only subunit whose downregulation affects neither the protein levels of the other eIF3 subunits (Figure 5G, Supplementary Figure S8G, and Table 3) nor the integrity of the eIF3 complex. All 11 remaining eIF3 subunits, as well as eIF3j, co-purify from eIF3d^KD^ cells (with either eIF3b and eIF3f baits) at levels unchanged from those observed with control cells (Figure 5H, Supplementary Figure S8H, Table 3; and Supplementary Figure S9E and F). Strikingly, despite the fully preserved integrity of the 11-subunit eIF3 complex in eIF3d^KD^ cells, they display some of the most severe defects we observe in both the rate of translation and cell growth (Figure 2).

### Increased protein turnover likely underlies the simultaneous downregulation of eIF3 subunits observed in cells where another eIF3 subunit has been knocked-down

Next we wished to investigate if the simultaneous downregulation of eIF3 subunits we observe when knocking down individual eIF3 subunits occurs because these subunits are degraded, either as a consequence of impaired eIF3 integrity or as a result of eIF3-specific mRNA translational control. To this end, we isolated total RNA from all polysomal fractions of a sucrose gradient prepared with whole cell extracts derived from Hela cells treated with either control siRNA or eIF3c siRNA and analyzed the polysomal association of mRNAs encoding each eIF3 subunit by RT-qPCR (Supplementary Figure S10). These experiments reveal no obvious difference in the polysomal distribution of mRNAs encoding subunits simultaneously downregulated in eIF3c^KD^ cells (d, k or l) compared with subunits whose levels are unaffected in these cells (b, g, i, f or m). To buttress these results, we further performed ribosome profiling in eIF3c^KD^ and eIF3f^KD^ cells and detected no change in the distribution of footprints over mRNAs of the eIF3 subunits, whose protein levels significantly decrease in these knock-downs (SW, Neelam Sen, Alan G. Hinnebusch and LSV, unpublished observations). These findings suggest that, at least in case of eIF3c^KD^ and eIF3f^KD^, the dominant mechanism underlying the simultaneous downregulation of eIF3 subunits in cells where another subunit within the complex has been knocked-down is increased protein turnover. This increased turnover is likely due to incomplete complex assembly and not to some peculiar translational control mechanism, which would have to be specific for selected sets of human eIF3 subunits.

## DISCUSSION

**Figure 6.**
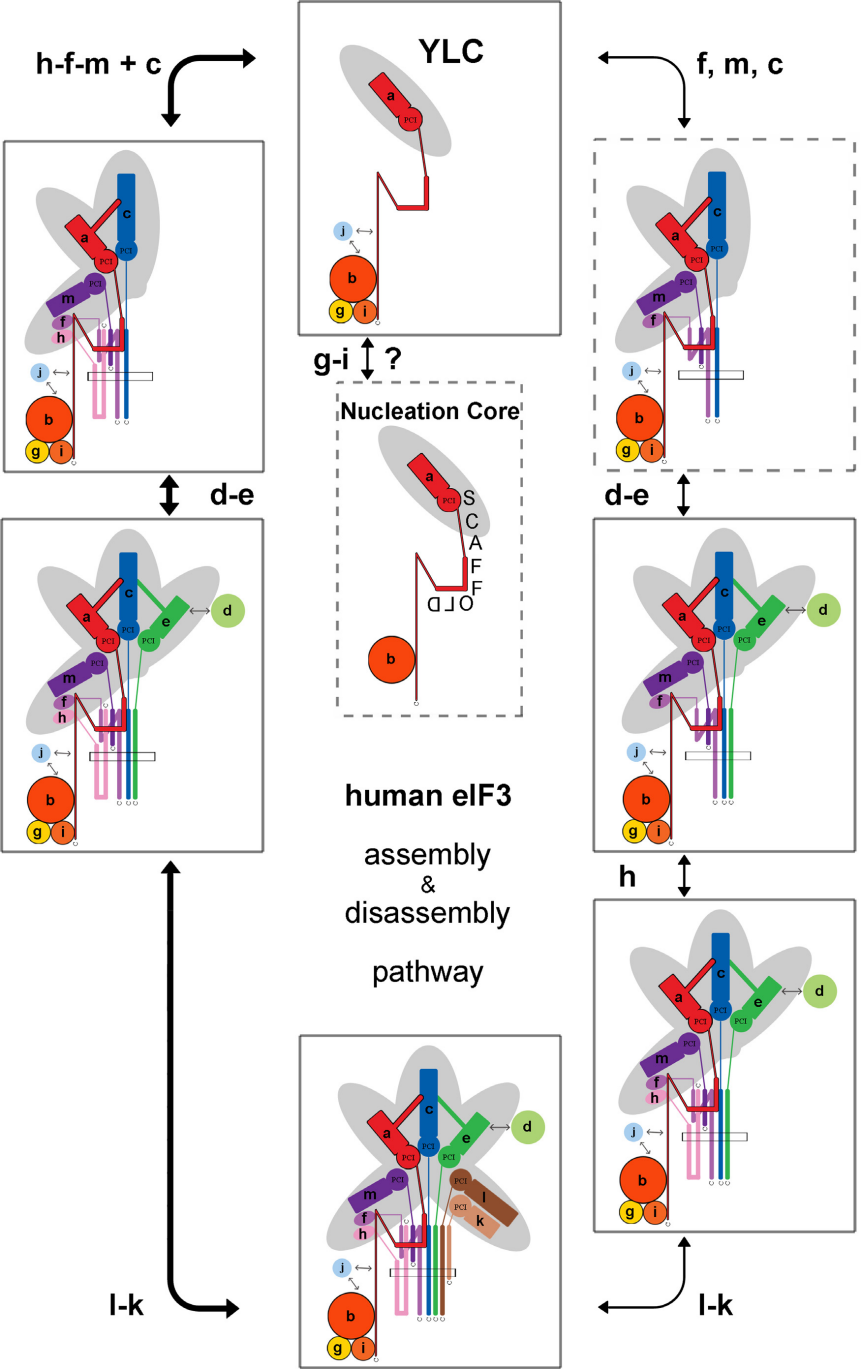
The assembly/disassembly pathways of the human eIF3 complex. The eIF3 subcomplexes that were observed in this or our previous study (39) are framed by a solid rectangle; subcomplexes that are based on our analysis predicted to form are framed by a dashed rectangle. The scaffold eIF3a subunit together with eIF3b first creates the eIF3 nucleation core. Subsequently, the subunits g and i join as a couple along the C-terminus (the spectrin domain (44)) of eIF3a to form the YLC (a question mark indicates that their attachment is not a strict prerequisite for the formation of the rest of eIF3 and that they may join eIF3 at any time of its assembly). Around the same time, the eIF3c subunit starts to form the octamer hand in hand with the h-f-m triangle; eIF3c cannot attach to YLC without eIF3f and/or m and *vice versa*, in contrast to *N. crassa* (47). Based on our ‘disassembly’ data, an alternative pathway is also depicted, where eIF3f, m and c join the complex first and the subunit h attaches to eIF3 further downstream; the resulting complex (framed by a dashed rectangle) is only our assumption. Subsequently eIF3e integrates into the complex bringing eIF3d along. Finally, subunits k and l attach as a pair to complete the assembly.

The results presented here extend our previous study (39), such that we have now monitored the effects of knocking down each individual subunit of eIF3 in HeLa cells on a broad array of mechanistic readouts: overall cell fitness, translation initiation rates, the integrity of the eIF3 complex, and the expression levels eIF3 subunits not directly targeted for downregulation by siRNA. Having catalogued these effects, we can now define for the first time the disassembly pathway of the human eIF3 complex (Figure 6). We suggest that the ordered disassembly of the eIF3 complex occurs as a consequence of the destabilization of several keystone interactions that hold the full complex together. Each one of these interactions contributes a varying degree of stability to the eIF3 complex. It is notable that many of these critical interactions lie either in the PCI/MPN domains of individual subunits, in the helical bundle of the octamer, or within the eIF3a subunit, itself the major scaffolding subunit within eIF3. The likely consequence of ordered eIF3 disassembly—triggered by targeted downregulation of specific eIF3 subunits—is the rapid degradation via the proteasome of those subunits no longer anchored within the eIF3 complex. Consistent with this interpretation, we observe no effect on the association of mRNAs encoding for the subunits of eIF3 with 80S couples in polysomes in either eIF3c^KD^ or eIF3f^KD^ cells, even for those subunits that are downregulated as a result of knocking down eIF3c or eIF3f. (Supplementary Figure S10 and unpublished observations). Based on our findings we propose and further discuss the assembly pathway for human eIF3. We believe this pathway likely mirrors the disassembly pathway but in reverse (Figure 6).

According to our siRNA experiments in HeLa cells, the eIF3 subunits k and l within the right leg and the eIF3-associated factor eIF3j are either dispensable for wild-type growth under normal conditions or capable of supporting normal translational rates at the residual levels (≤20% of WT) we observe in knock-down cells (Figure 2 and (39)). We strongly favor the first interpretation for several reasons. First, neither eIF3k nor eIF3l exist in *Schizosaccharomyces pombe*, whose eIF3 complex otherwise more closely resembles the mammalian complex than the budding yeast complex (45,46). Second, neither of these subunits is essential in *N. crassa* (47) (nor is eIF3j in *S. cerevisiae* (38)). Finally, it is unlikely that these subunits are both indispensable and yet their absence from ∼80% of eIF3 complexes (Figure 3) would not affect the cell's ability to satisfy the protein synthesis demands of rapid proliferation. We observe that the protein levels of eIF3k and l depend strictly on each other, suggesting that they form a dimer that precedes their assembly within the eIF3 complex, most probably during the last step of eIF3 assembly *in vivo* (Figure 6). In support of this, the association of the eIF3k:eIF3l dimer with eIF3 requires prior recruitment of eIF3e and eIF3h to the complex; knocking down eIF3h or eIF3e results in the strong and simultaneous downregulation of both eIF3k and l (Figures 3 and 4). Knock-down of eIF3e also robustly reduces the levels of eIF3d (see below). In contrast, eIF3d^KD^ does not affect the levels of either eIF3k or eIF3l (Figure 5). A similar interdependence between the eIF3k and l subunits *in vivo* was observed in *N. crassa* (47). The *in vivo* dependence of eIF3l (and thus in turn also of eIF3k) on the eIF3e subunit can be explained by the fact that the PCI domains of these subunits directly interact with each other (32). This is not the case, however, for eIF3h, which resides on the other side of the PCI arc. This suggests that eIF3h forms a supportive joint with eIF3k and/or l within the 7-helix bundle of the octamer (32). In fact, while this article was in preparation, Smith and colleagues (40) demonstrated the importance of interactions within the helical bundle for the integrity of eIF3 in *N. crassa*. Taken together, these observations suggest that while the eIF3k and l subunits are dispensable during normal conditions, they may nonetheless play an important regulatory role under stress conditions or during development or aging, as recently proposed (48).

Upon knocking down the eIF3h subunit of the left leg, which strongly reduced the levels of both eIF3k and l and more modestly downregulated several other eIF3 subunits, we observe two unique eIF3 complexes: 1) the YLC bound by the eIF3-associated factor eIF3j (YLC+j) and 2) the 9-subunit complex+eIF3j lacking eIF3h, k and l, which occurs at approximately half the abundance of the YLC+j complex (Figure 4). That eIF3h knockdown precipitates the dissolution of eIF3 into these distinct subcomplexes indicates that eIF3h plays an important supporting role in stabilizing the eIF3 octamer. Because eIF3h knock-down only modestly affects growth and initiation rates (Figure 2), it is highly likely that the reduced cellular levels we observe for the 9-subunit complex are sufficient to support life-sustaining rates of protein synthesis. In support of this interpretation, eIF3h was recently shown to be non-essential in HEK293 cells (the eIF3h deletion did not display an obvious phenotype), and previously in both *S. pombe* (its deletion affected spore formation) and in *N. crassa* (reduced aerial hyphae) (40,47,49).

Importantly, because we observe the YLC+j complex upon knocking down several distinct eIF3 subunits (eIF3c, e, f, h, and m) (Figures 3 and 4 and (39)), this complex appears to assemble completely independently of the octamer, perhaps as soon as the scaffolding eIF3a subunit interacts with eIF3b to form the nucleation core of the eIF3 complex (Figure 6). In fact, we find that formation of this eIF3-nucleation core is the key prerequisite, not only for the formation of the YLC+j complex, but for the octamer as well (Figure 5B). Thus, we propose that once the a-b dimer forms, both the YLC and the octamer independently nucleate around their interacting partners within this nucleation core, eIF3b and eIF3a respectively. Consistent with this model, our preliminary yeast ribosomal profiling data indicate that eIF3a/TIF32 interacts with eIF3b/PRT1 co-translationally as soon as the extreme N-terminal RRM domain of PRT1 folds (SW, Nick Ingolia and LSV, unpublished observations). Moreover, reduced expression of any one subunit of the YLC b-g-i trimer led to markedly reduced expression of all other subunits (with the exception of the eIF3-associated factor eIF3j) and conferred severe growth defects (Figures 2 and 5).

Elsewhere, the presence of eIF3g and i within the eIF3 complex strictly depend on each other, and so does their expression. And yet, downregulation of eIF3g has a more pronounced effect on the protein stability of eIF3i than *vice versa*. We suggest that, like eIF3k and l, eIF3g and i can form a dimer that subsequently associates with eIF3b during assembly of the YLC. However, this dimer is likely not strictly required for the formation of the rest of the eIF3 complex (Figure 6). In support of this proposal, the steady state levels of both eIF3g and i are the least sensitive among the eIF3 subunits to eIF3b knock-down (Figure 5). Despite the fact that knocking down each of these two YLC subunits does not prevent the formation of the eIF3 complex, their downregulation does partially destabilize the connection between eIF3a and eIF3b. Consistent with this, a human eIF3 complex missing the g and i subunits can also form *in vitro* (33). Interestingly, a core complex lacking TIF34 (eIF3i) and TIF35 (eIF3g) also forms in budding yeast cells. This subcomplex is capable of promoting ternary complex and mRNA recruitment to 40S ribosomes *in vitro*, though subsequent experiments have suggested these subunits are important, both for ternary complex stabilization and the recruitment of a natural mRNA (42,43). In fact, the critical role of TIF34 and TIF35 in translation initiation most probably lies in their stimulation of ribosomal scanning (50,51). We postulate that eIF3g and i stabilize the assembly of the evolutionary conserved YLC, which in turn fortifies the connection between the YLC and the octamer that is mediated mainly by the eIF3b and eIF3a nucleation core. Interestingly, eIF3i is the lone subunit found to have a related Archaean homologue and thus is likely the most conserved of the eIF3 subunits, which may indicate that it indeed plays a critical role within eIF3 (36).

It is surprising that we do not detect a YLC complex containing the eIF3c subunit (NIP1 in yeast), despite eIF3c being well expressed in eIF3e^KD^, f^KD^ and m^KD^ cells. Yeast eIF3c (NIP1) forms a stable trimer with eIF3a (TIF32) and eIF3b (PRT1), both *in vitro* and *in vivo* (42,43). Moreover, human eIF3a and eIF3c form a stable dimer *in vitro* (33), and the YLC+eIF3c pentameric complex has previously been detected by mass spectrometry (7). Nonetheless, we have previously shown that the YLC lacking eIF3c is able to perform the basic functions of eIF3 in HeLa and HEK293 cells, though with expectedly reduced efficiency (39). Thus, the YLC likely represents a minimal functional unit of the eIF3 complex partially capable of promoting the core events of the initiation pathway, such as the binding of ternary complex and mRNA to the 40S ribosome. This minimal complex may further contribute to scanning, though processive scanning appears to require eIF3c (39).

From the pattern of eIF3 subcomplexes formed upon knocking down either eIF3c or eIF3e, we are able to deduce that eIF3c is required to closely collaborate with eIF3a to connect the subunits composing the left hand (f, h, m) to the YLC, with the eIF3e subunit of the right hand further supporting this assembly step (Figure 3 and (39)). eIF3e undoubtedly further serves to connect the eIF3d subunit to the rest of eIF3, as reported previously (32). The eIF3e and eIF3d subunits further appear to form a stable dimer prior to their joining the remainder of the complex (40), similar to the eIF3g:eIF3i and eIF3k:eIF3l dimers. Interestingly, the 8-subunit complex (i-g-b-**a**-c-f-h-m) we observe upon knocking down eIF3e is one of the two distinct eIF3 complexes shown to naturally occur in *S. pombe* (45,46). The conservation of this complex, together with recent studies demonstrating the importance of the helical bundle in *N. crassa* (40) and a recent structure of mammalian eIF3 (32), suggest that the helical bundle formed by the octamer subunits represents, like the mutual contacts between the PCI and MPN domains of eIF3a and the f-h-m trimer, another keystone interaction network that cements the attachment of the left leg to the rest of the eIF3 body.

Turning to the role of eIF3f and eIF3m, our results demonstrate that in the absence of these two subunits of the left leg, the octamer cannot form. This formation is blocked despite the fact that the protein levels of both arms (subunits eIF3a and e) and the eIF3 head (eIF3c) remain high in eIF3f^KD^ and eIF3m^KD^ cells (Figure 4). This implies that, while the a-c dimer, which was originally proposed to function as the eIF3 nucleation center, can form *in vitro* (33), it cannot form *in vivo* unless at least these left leg subunits—eIF3m and eIF3f—are well expressed (Figures 4 and 6—dashed rectangle). Because eIF3e is dispensable for the formation of the minimal a-c-f-h-m subcomplex of the octamer (Figure 3), we propose that the i-g-b-**a**-c-f-h-m YLC-octamer subcomplex represents one of the major intermediates of the eIF3 assembly pathway (Figure 6). The fact that eIF3m is missing from the eIF3 complex in all protists (36) suggests that, of these two subunits, it is eIF3f that plays a more critical role in stabilizing the a–c dimer and its connection to the YLC.

Perhaps the most striking result we obtain is upon knocking down eIF3d, which has no impact on the expression levels of other eIF3 subunits nor on the integrity of eIF3 *in vivo* (Figure 5), but nonetheless confers severe defects in growth and translation rates (Figure 2). The eIF3d subunit is attached to the eIF3 holocomplex via eIF3e, which is absent in *S. cerevisiae*, but is nonetheless found in the majority of other studied organisms. eIF3e is essential in *N. crassa* (52) and in *S. pombe*, where two distinct eIF3 complexes have been observed; the presence of eIF3d (and eIF3e) distinguishes a subcomplex thought to be responsible for the translation of a restricted set of mRNAs; the other complex, which contains the full complement of eIF3 subunits is thought to promote the translation of all mRNAs (45,46). While it is indeed possible that the loss of eIF3d may result in an intact but functionally inactive complex, it is tempting to speculate that the essential character of eIF3d does not lie in its indispensability for the canonical roles of eIF3 in general translation, but instead in promoting the translation of specific mRNAs encoding proteins with vital cellular roles. Indeed, eIF3d was recently identified as a non-canonical cap binding protein that in complex with eIF3 drives cap-dependent but eIF4F-independent expression of a specific subset of mRNAs (14,53). In fact, such a role for eIF3d, and perhaps some other eIF3 subunits, might explain the differential severity of growth and initiation phenotypes observed in eIF3c^KD^ and eIF3e^KD^ *versus* eIF3f^KD^ and eIF3m^KD^ cells. Future experiments are required to explain precisely why eIF3d is essential.

Our eIF3 knock-down analysis produced two patterns of expression dependencies among the eIF3 subunits that appear to reflect the ability of the remaining subunits to form subcomplexes and thus escape the proteolysis that our results suggest is the fate of unbound subunits. The first of these two patterns implies that eIF3g impacts the steady state levels of eIF3i, which in turn controls eIF3b, followed by a, c, e, and finally (in no particular order) the trio d, k and l. In short: g>i>b>a>c>e>d,k,l. The second pattern, which differs from the first in the subunits that are affected by eIF3a, suggests that eIF3a in addition impacts the f-m subunits, followed by eIF3h and finally the k-l dimer (a>f,m>h>k,l). The expression of the eIF3-associated factor eIF3j was unaffected by any of the eIF3 knock downs; eIF3j was always found in complexes co-immunoprecipitated by anti-eIF3b (Figures 3–5). This is consistent with the known interaction between eIF3j/HCR1 and the RRM domain of eIF3b/PRT1, both in humans and in *S. cerevisiae* (9,11).

The implications of our results on human disease are underscored by the fact that altered expression levels of the eIF3 subunits has been observed in various types of cancer (1). Our results demonstrate that these perturbations to the relative balance of eIF3 subunits lead to the formation of partial eIF3 subcomplexes that are associated with defects in the rate of translation and cell fitness and thus begin to illuminate the potential role of eIF3 in cancer and other human diseases.

## Supplementary Material

SUPPLEMENTARY DATA
