# Supplementary material for: Human eIF3b and eIF3a serve as the nucleation core for the assembly of eIF3 into two interconnected modules: the yeast-like core and the octamer
Source: Nucleic Acids Res. 2016 Oct 19;44(22):10772–88. doi: 10.1093/nar/gkw972 (PMC5159561; doi:10.1093/nar/gkw972)
Supplement: SUPPLEMENTARY DATA [file supp_44_22_10772__index.html]

Human eIF3b and eIF3a serve as the nucleation core for the assembly of eIF3 into two interconnected modules: the yeast-like core and the octamer — Human eIF3b and eIF3a serve as the nucleation core for the assembly of eIF3 into two interconnected modules: the yeast-like core and the octamer — SUPPLEMENTARY DATA 

# Human eIF3b and eIF3a serve as the nucleation core for the assembly of eIF3 into two interconnected modules: the yeast-like core and the octamer

## SUPPLEMENTARY DATA

- SUPPLEMENTARY DATA
