# Supplementary material for: Human eIF3b and eIF3a serve as the nucleation core for the assembly of eIF3 into two interconnected modules: the yeast-like core and the octamer
Source: Nucleic Acids Res. 2016 Oct 19;44(22):10772–88. doi: 10.1093/nar/gkw972 (PMC5159561; doi:10.1093/nar/gkw972)
Supplement: SUPPLEMENTARY DATA [file supp_gkw972_nar-02552-a-2016-File008.pdf]

Supplementary Material

Supplementary Figures and Figure legends

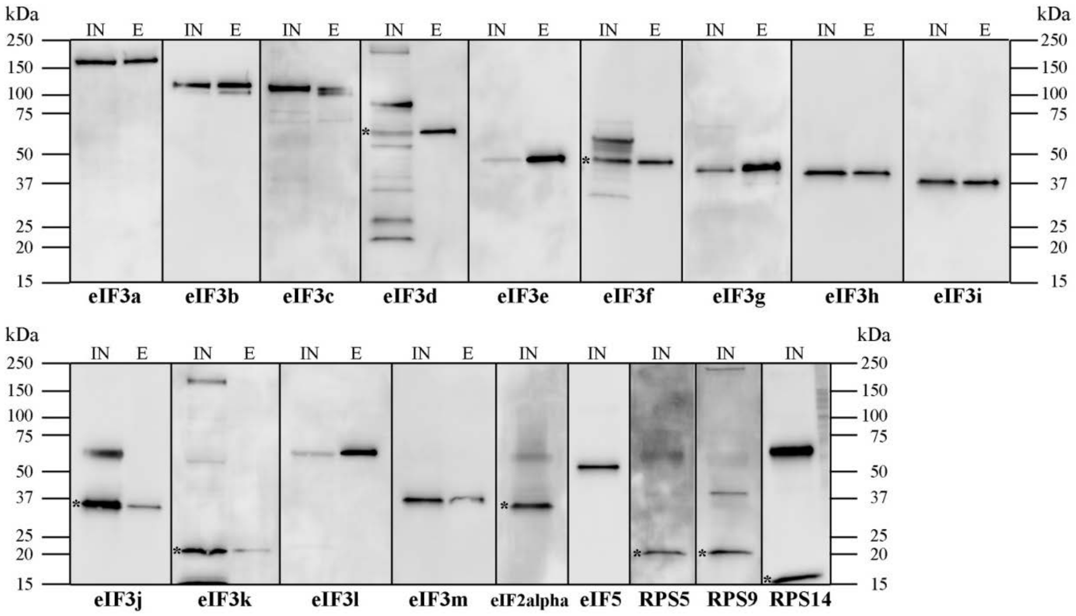

Supplementary Fig. 1. Overview of the antibodies used in this study. The Western signals obtained from all antibodies used throughout this study are shown individually on an uncropped blot. The gels were loaded either with 60µg of total protein from whole cell extracts derived from HeLa cells treated with non-targeting siRNA (IN) or with 10% of the elution fraction from the co-immunoprecipitation experiments performed with anti-eIF3b antibodies (E). The size markers are indicated. The specificity of each band (marked by an asterisk where needed) was determined by 1) its mobility at the expected position on the SDS-PAGE as predicted by its molecular weight, 2) the fact that it specifically co-immunoprecipitates with the rest of eIF3 (see our eIF3b and eIF3f Co-IP experiments here and in (1), and 3) the fact that it specifically co-sediments with PICs in sucrose gradients (1). For a list of the antibodies please see Table S2.

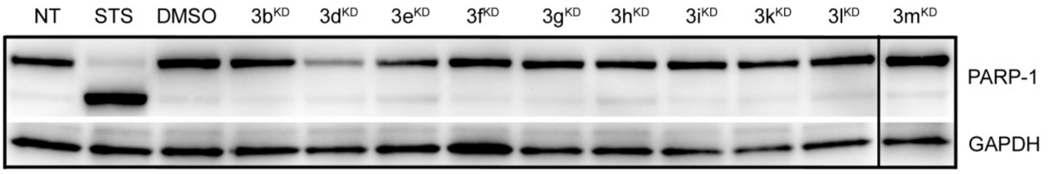

Supplementary Fig. 2. Cells knocked down for individual eIF3 subunits are not apoptotic at the time of performing the experiments. All knock-downs were checked for the presence of cleaved PARP-1, an indicator of apoptosis, by Western blotting, 3 days after siRNA transfection. GAPDH was used as loading control. NT: cells treated with non-targeting siRNA; STS: cells treated with 1µM Staurosporine, an inducer of apoptosis, for 4h; DMSO: cells treated with DMSO, the dissolvent of Staurosporine. The level of expression of PARP-1 in the 3d^{KD} appears to be reduced, which might indicate that PARP-1 is partially downregulated in the eIF3d knocked down cells; this effect was, however, not examined any further in this study.

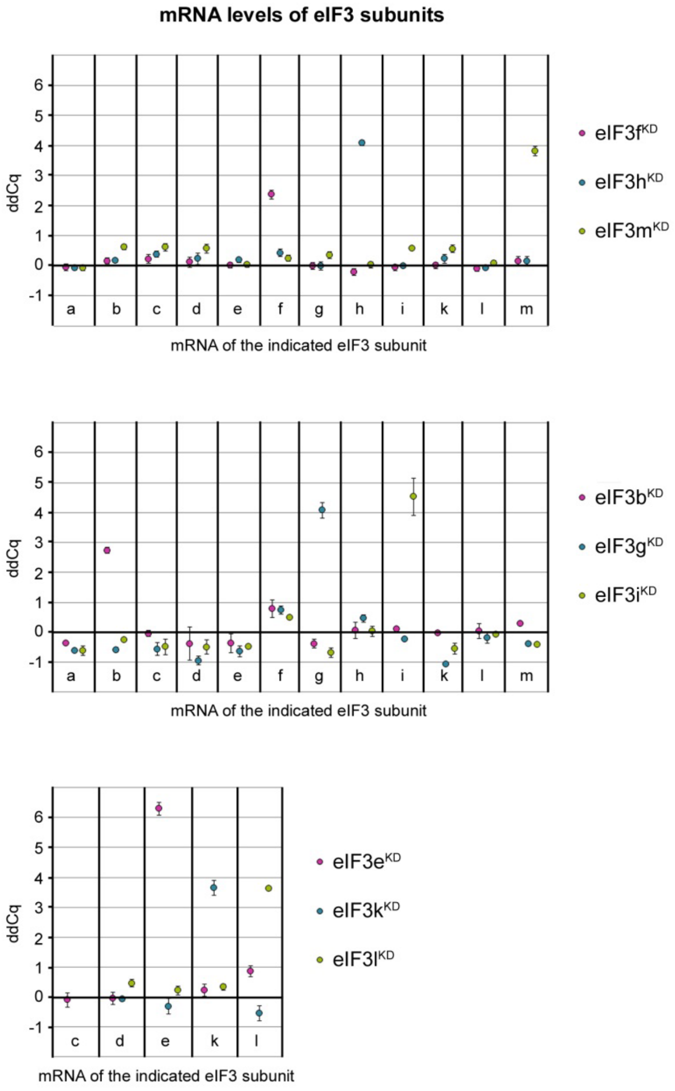

Supplementary Fig. 3. The mRNA levels of all eIF3 subunits upon knock down of an indicated subunit on target were assessed by quantitative PCR 3 days post-transfection. Plots represent the results of three independent experiments ± SD. The ddCq value displays the threshold cycle normalized to the reference gene B2MG and to NT control cells. The ddCq values are in log2 scale. A ddCq of 0 indicates no change to NT cells; ddCq = 1 or = -1 indicates a drop of 50% or doubling compared to NT cells, respectively.

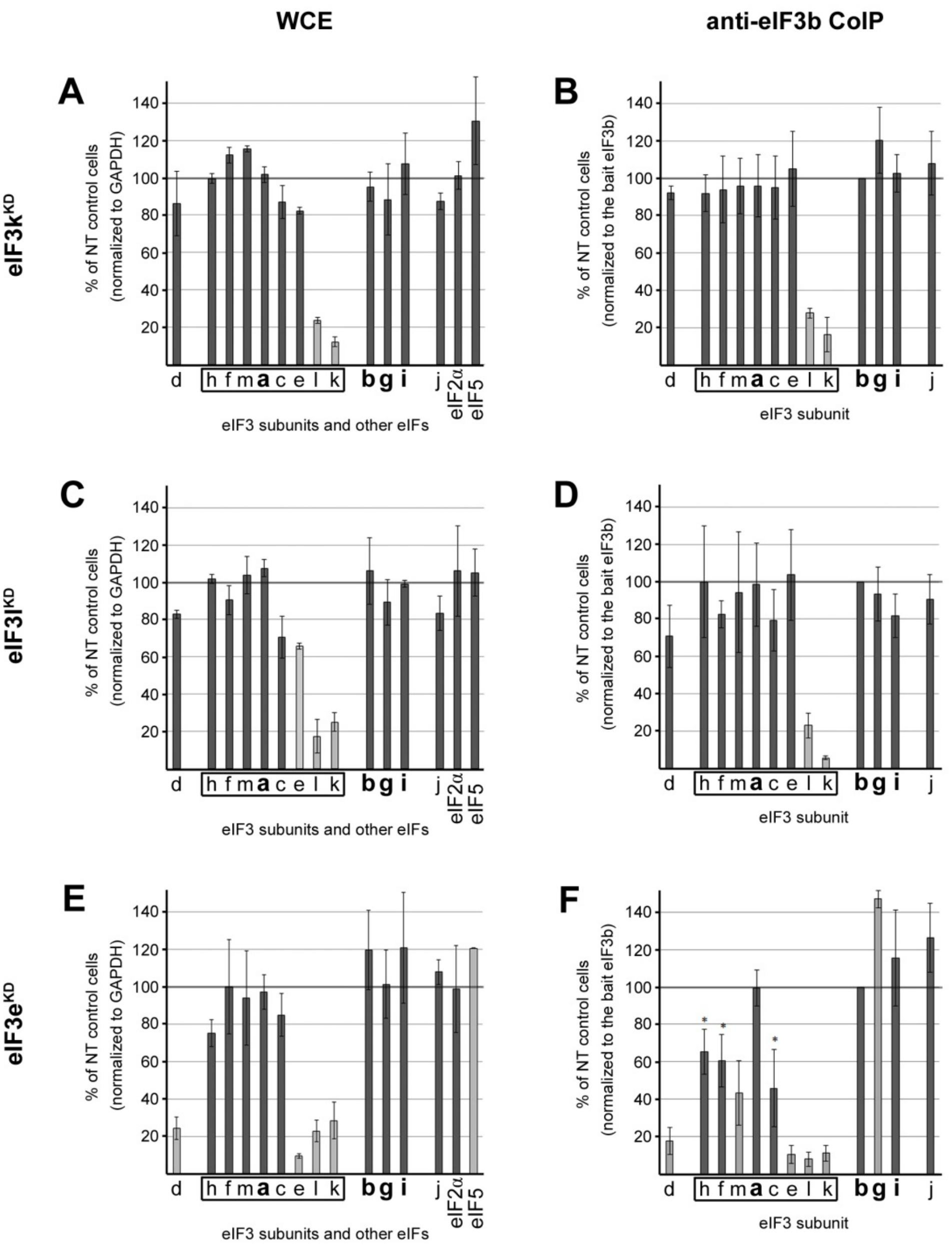

Supplementary Fig. 4. The octameric right leg subunits eIF3k and eIF3l impact their own expression and are dispensable for the integrity of the rest of eIF3, and the octameric right arm subunit eIF3e stabilizes binding of the right leg subunits (k, l) and eIF3d to the octamer, as well as the octamer attachment to the YLC. Quantification of experiments shown in Fig. 3. Quantified signals were normalized to NT control cells and to GAPDH or the bait eIF3b for the total protein levels or the CoIP analysis. The average of at least four experiments is shown ±SD. The rectangle encloses all eIF3 subunits forming the PCI/MPN octamer. eIF3 subunits comprising the yeast-like core (YLC) are highlighted in bold. A list of all values can be found in Table 1. Dark grey bars indicate no significant change compared to NT control cells while light grey bars indicate a significant change at $p \leq 0.01$ (one sample *t*-test). (F) Asterisks indicate a significant change at $p \leq 0.05$; please also note the signals for eIF3h, f, m, and c show no significant differences

within this group; however, they differ significantly when compared to the group containing eIF3d, e, k, and l, or to the group featuring eIF3a, b, g, and i (*t*-test, $p \leq 0.05$).

A

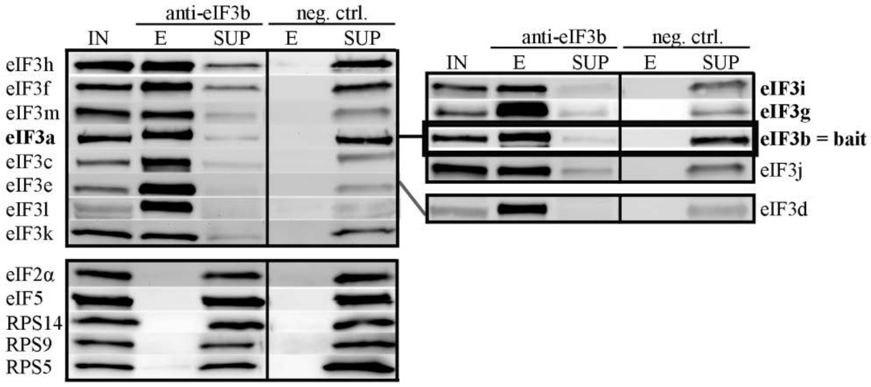

B

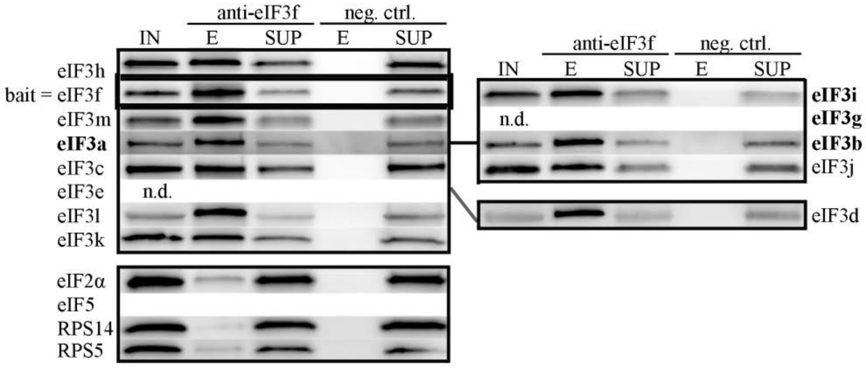

Supplementary Fig. 5. Evidence of specificity of the anti-eIF3b and anti-eIF3f CoIP assays. (**A**) Anti-eIF3b and (**B**) anti-eIF3f co-immunoprecipitation experiments were performed with WCE of HeLa cells 3 days after transfection with non-targeting siRNA. Input (IN), eluate (E) and supernatant (SUP) fractions were subjected to Western blotting with antibodies indicated on both sides; in (**A**) 8% of IN and SUP and 25% of E, and in (**B**) 5% of IN and SUP and 20% of E were loaded. For the negative control (neg. ctrl.) the beads without antibodies were used. Octameric eIF3 subunits are arranged at the left-hand side and the non-octameric subunits are shown at the right-hand side. The YLC subunits are highlighted in bold. In (**B**) the signal of our anti-eIF3e and anti-eIF3g antibodies could not be clearly visualized because of the heavy chain of eIF3f antibodies of the rabbit origin migrating at the same size (n.d. = not determined). The band seen in the E lane with the eIF5 strip is a routinely observed cross-reaction.

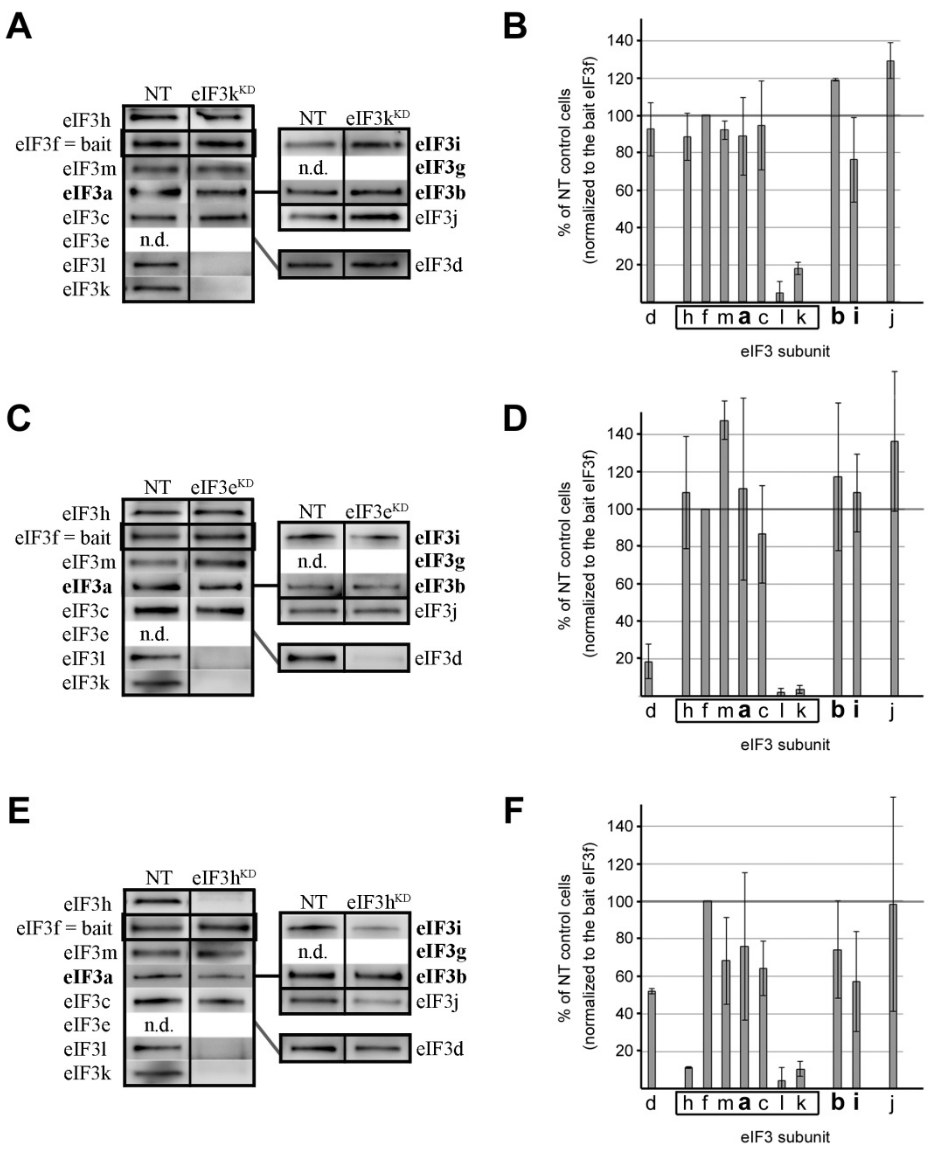

Supplementary Fig. 6. The eIF3f-CoIP assays performed in the eIF3k^{KD} (A), eIF3e^{KD} (C), and eIF3h^{KD} (E) cells to examine the integrity of the eIF3 octamer. Similar to Fig. 5B, quantifications of at least five experiments ±SD is shown for eIF3k^{KD} (**B**), eIF3e^{KD} (**D**) and eIF3h^{KD} (**F**); quantified signals were normalized to NT control cells and to the bait eIF3f. The rectangle encloses all eIF3 subunits forming the PCI/MPN octamer. eIF3 subunits comprising the yeast-like core (YLC) are highlighted in bold. The signal of our anti-eIF3e and anti-eIF3g antibodies could not be clearly visualized because of the heavy chain of eIF3f antibodies of the rabbit origin migrating at the same size (n.d. = not determined).

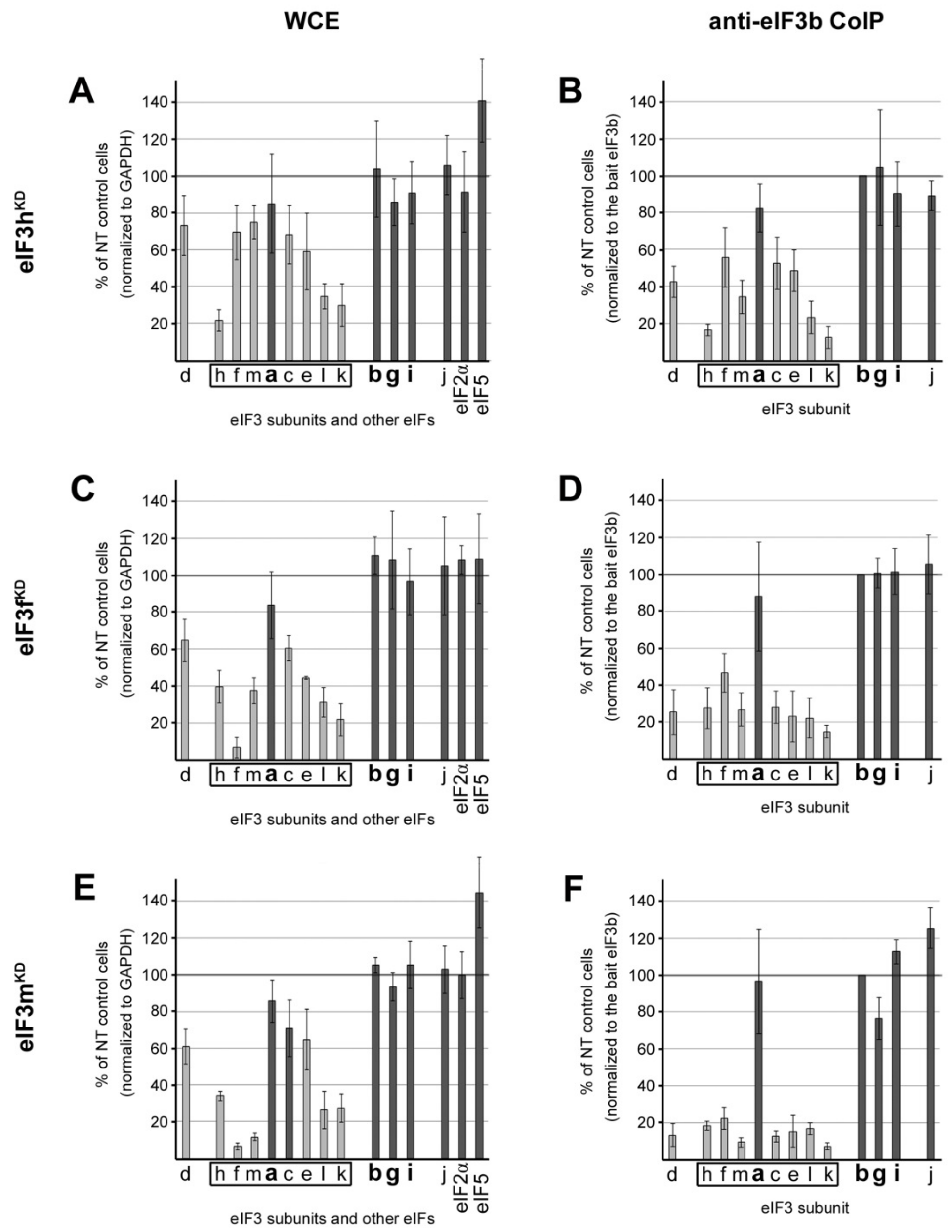

Supplementary Fig. 7. The octameric left leg subunits eIF3f and m represent, together with eIF3c and eIF3a, the key building blocks of the PCI/MPN octamer. Quantification of experiments shown in Fig. 4. Quantified signals were normalized to NT control cells and to GAPDH or the bait eIF3b for the total protein levels or the CoIP analysis. The average of at least five experiments is shown ±SD. The rectangle encloses all eIF3 subunits forming the PCI/MPN octamer. eIF3 subunits comprising the yeast-like core (YLC) are highlighted in bold. A list of all values can be found in Table 2. Dark grey bars indicate no significant change compared to NT control cells while light grey bars indicate a significant change at $p \leq 0.01$ (one sample *t*-test). (B) Please note that the signals for eIF3d, f, m, c, and e show no significant differences within this group (with the exception of eIF3m); however, they differ significantly when compared to the group containing eIF3h, l, and k (except for eIF3m to l), or to the group featuring eIF3a, b, g, and i (*t*-test, $p \leq 0.05$).

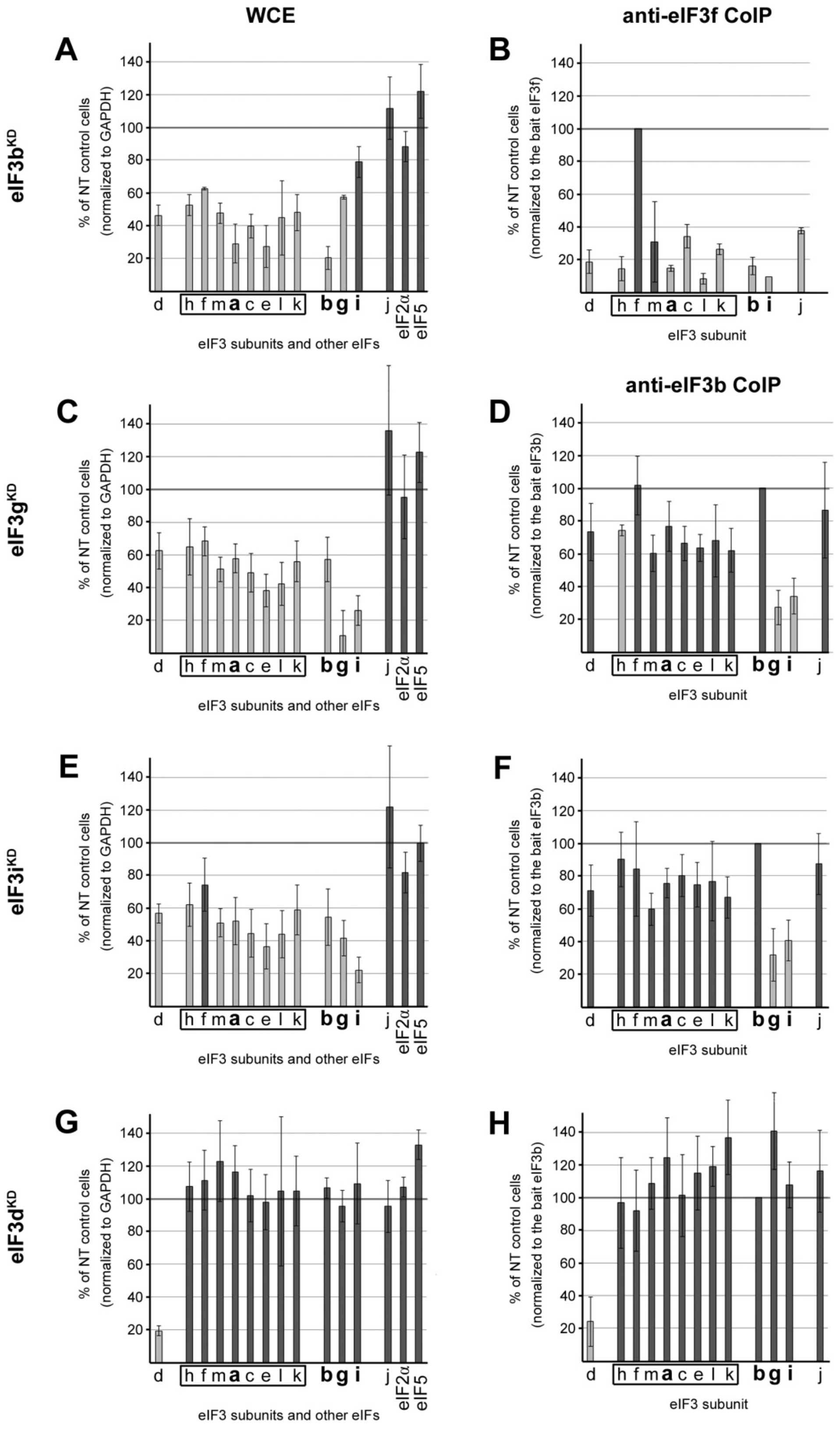

Supplementary Fig. 8. The YLC subunits eIF3b, g and i are required for a stable formation of the eIF3 holocomplex, whereas eIF3d is dispensable for stable complex formation. Quantification of experiments shown in Fig. 5. Quantified signals were normalized to NT control cells and to GAPDH (panel **A, C, E, G**) or the bait eIF3b (panel **D, F** and **H**) or eIF3f (panel **B**) for the total protein levels or the

CoIP analysis. The average of at least five experiments is shown ±SD. The rectangle encloses all eIF3 subunits forming the PCI/MPN octamer. eIF3 subunits comprising the yeast-like core (YLC) are highlighted in bold. A list of all values can be found in Table 3. Dark grey bars indicate no significant change compared to NT control cells while light grey bars indicate a significant change at $p \leq 0.01$ (one sample *t*-test).

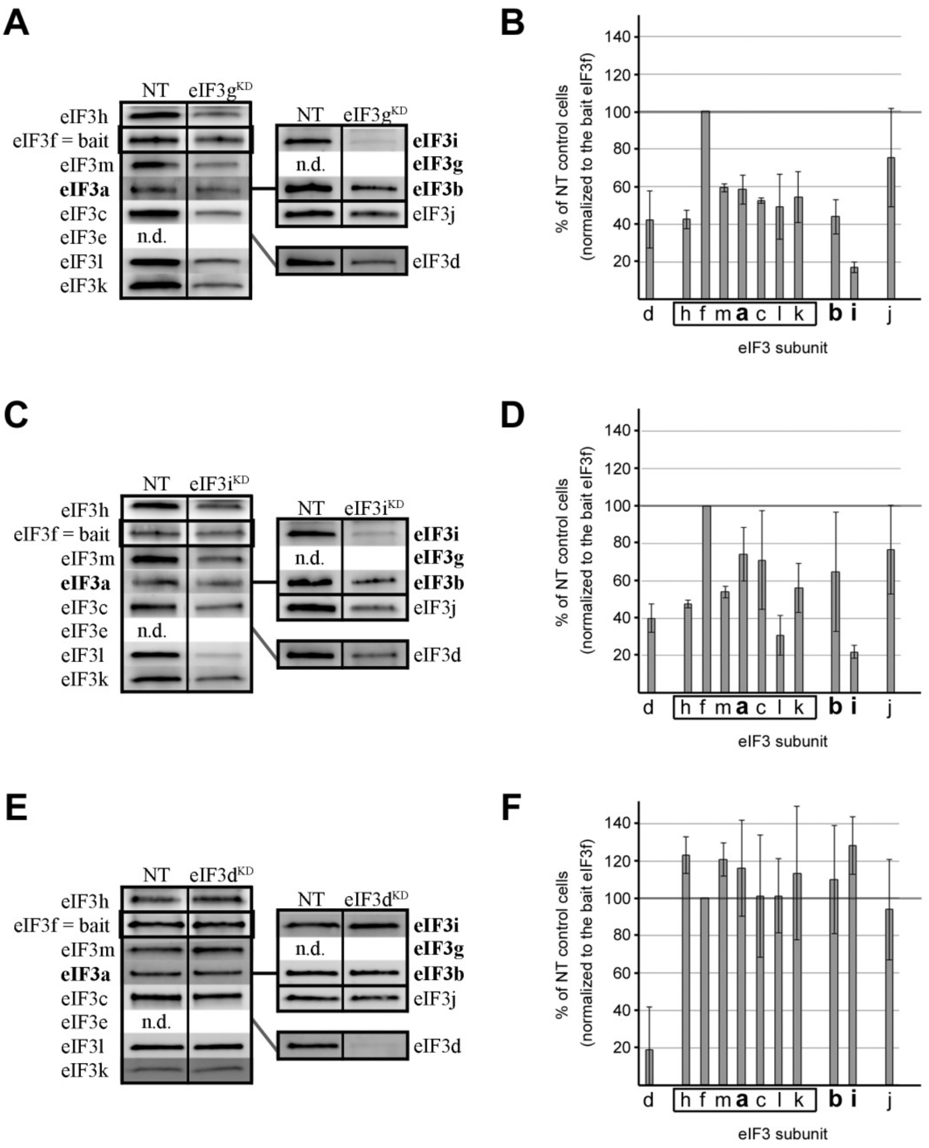

Supplementary Fig. 9. The eIF3f-CoIP assays performed in the eIF3g^{KD} (A), eIF3i^{KD} (C), and eIF3d^{KD} (E) cells to examine the integrity of the eIF3 octamer. Similar to Fig. 5B, quantifications of at least four experiments ±SD are shown for eIF3g^{KD} (**B**), eIF3i^{KD} (**D**) and eIF3d^{KD} (**F**); quantified signals were normalized to NT control cells and to the bait eIF3f. The rectangle encloses all eIF3 subunits forming the PCI/MPN octamer. eIF3 subunits comprising the yeast-like core (YLC) are highlighted in bold. The signal of our anti-eIF3e and anti-eIF3g antibodies could not be clearly visualized because of the heavy chain of eIF3f antibodies of the rabbit origin migrating at the same size (n.d. = not determined).

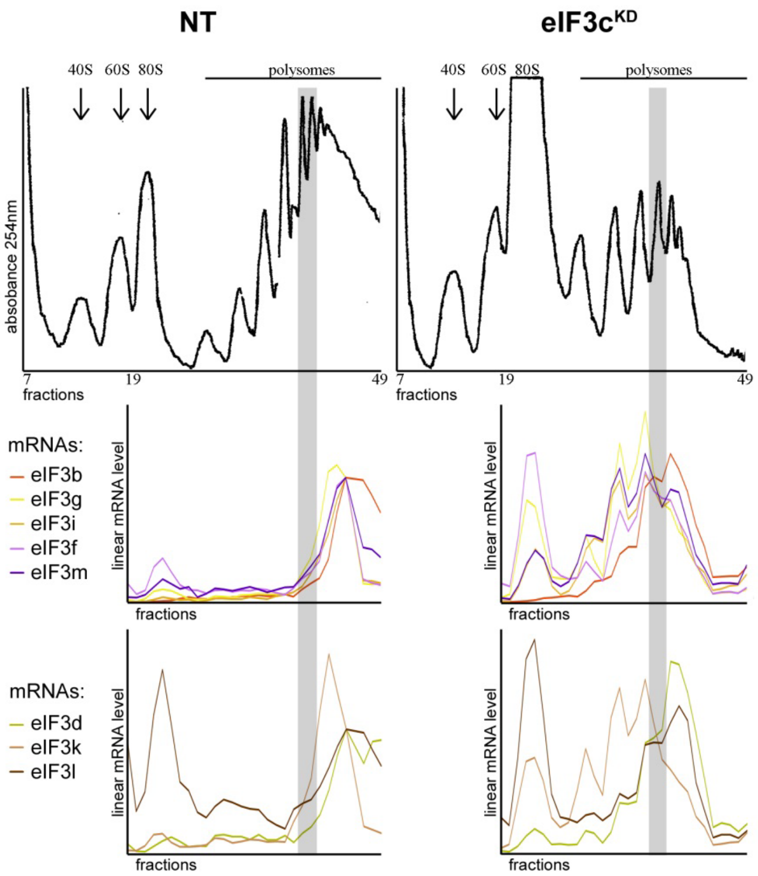

Supplementary Fig. 10. All mRNAs encoding the eIF3 subunits in the eIF3c^{KD} remain associated with polysomes. Total RNA was isolated from 49 polysomal fractions of a sucrose gradient prepared with WCE of Hela cells treated either with control siRNA (NT) or eIF3c siRNA (eIF3c^{KD}), and the polysomal association of mRNAs encoding selected eIF3 subunits was analyzed by RT-qPCR. Polysomal profiles are shown in the upper panels. The middle panels depict RT-qPCR results obtained for mRNAs of eIF3 subunits that are expressed normally in the eIF3c knock down; i.e. their protein levels are not affected by this knock-down (1). RT-qPCR results of eIF3 subunits that are co-downregulated upon eIF3c knock down are then shown in the lower panels. For better orientation, the most abundant polysomes are marked by the grey rectangle. To enable depiction of all curves in one diagram, these had to be adjusted to the eIF3b mRNA in the fraction 44 or 38 for NT or eIF3c^{KD}, respectively.

Supplementary references

1. Wagner, S., Herrmannova, A., Malik, R., Peclinovska, L. and Valasek, L.S. (2014) Functional and Biochemical Characterization of Human Eukaryotic Translation Initiation Factor 3 in Living Cells. *Mol Cell Biol*, **34**, 3041-3052.

Supplementary Tables

Table S1. siRNAs used in the study.

ON-TARGETplus siRNA	cat #
eIF3a	L-019534-00
eIF3b	L-019196-00
eIF3c	L-009036-00
eIF3d	L-017556-00
eIF3e	L-010518-00
eIF3f	L-019535-00
eIF3g	L-019533-00
eIF3h	L-003883-00
eIF3i	L-019531-00
eIF3j	L-019532-00
eIF3k	L-020216-02
eIF3l	L-020949-01
eIF3m	L-016219-01
Non-targeting	D-001810-03

Table S2. Antibodies used in the study.

antibody	source
eIF3a	Cell Signalling # 2538
eIF3b	Thermo Scientific # PA5-23278
eIF3c	Santa Cruz # sc-28858
eIF3d	kind gift of Dr. Imataka
eIF3e	Abcam # ab36766
eIF3f	kind gift of Dr. Imataka
eIF3g	Thermo Scientific # PA5-25261
eIF3h	Cell Signalling # 3413
eIF3i	Sigma # HPA029939
eIF3j	Santa Cruz # sc-50356
eIF3k	Abcam # 85968
eIF3l	Bethyl # A304-753A-T
eIF3m	Sigma # HPA031063
GAPDH	Thermo Scientific # PA1-987
eIF2α	Santa Cruz # sc-133132
eIF5	Santa Cruz # sc-282
PARP	Cell Signalling # 9542P
RPS5	Sigma # HPA055878
RPS9	Thermo Scientific # PA5-13569
RPS14	Santa Cruz # sc-68873
eIF3b (for CoIP)	Santa Cruz # sc-16377

Table S3. Primers used in the study.

name	5'- 3' sequence
eIF3a*	TAAGAAACCAGCTGACAGC
eIF3a R*	CTTTCTCTTGCAGTATATGAGC
eIF3b*	TGTGAAAGGTACCTGGTGAC
eIF3b R*	AATAGGCCAATGGGCTGAG
eIF3c	GAGTCAGTGCTGCAACTTTC
eIF3c R	TAGCTTTGTCCTCCCGTTTC
eIF3d	CCAACCCAAACCCGTTTGTG
eIF3d R	TCTTCAGCTCCGTGGCAATG
eIF3e	CTGGTTCCAGCAACAGATAG
eIF3e R	GTGGCTGATAAAGGAAGAGG
eIF3f	ACGAGTACTACAGCCGAGAG
eIF3f R	TGCTGCAAGTCACTTGAGAG
eIF3g	TCGGGAACTCAGAGTTTGAC
eIF3g R	TCTCCTTCTCGCCAGTAGAC
eIF3h*	GCTGACTTTGATGAAGTCCA
eIF3h R*	ATGTGGACTGATACCAGCC
eIF3i*	CATCATGTTCTCCACGGAC
eIF3i R*	CATTGTTGTCAATCTGGCTC
eIF3j	GAGGACGTCAAGGATAAC
eIF3j R	TTCGAGGTCTGACTCTTC
eIF3k	GTTCAACCCAGCCTTCTTTC
eIF3k R	TCGGACAGAGTCTTCAAAGC
eIF3l	ATTGCCCTCACGATGTACCC
eIF3l R	CTGCGGATGGTTGAAAGCTG
eIF3m*	AAGAAGATCAGGCTGCTGAG
eIF3m R*	GTCCACCTTCCGAGTTCTC
B2MG	GTATGCCTGCCGTGTGAACCATG
B2MG R	CAAATGCGGCATCTTCAAACCTCC
yeast RPL41	CGAAATGAGAGCCAAGTGG
yeast RPL41 R	ATGCAATTTAGATCCATTATGAGG

*primers' sequences were obtained from the database GETPrime, available at http://bbcftools.epfl.ch/getprime
